# The genetic landscape of autism spectrum disorder in an ancestrally diverse cohort

**DOI:** 10.1038/s41525-024-00444-6

**Published:** 2024-12-04

**Authors:** Ashlesha Gogate, Kiran Kaur, Raida Khalil, Mahmoud Bashtawi, Mary Ann Morris, Kimberly Goodspeed, Patricia Evans, Maria H. Chahrour

**Affiliations:** 1 Eugene McDermott Center for Human Growth and Development, University of Texas Southwestern Medical Center, Dallas, TX 75390 USA; 2 Department of Biotechnology and Genetic Engineering, Faculty of Science, University of Philadelphia, Amman, Jordan; 3 Department of Psychiatry, Jordan University of Science and Technology, King Abdullah University Hospital, Ramtha, Jordan; 4 UT Southwestern and Children’s Health Center for Autism Care, Children’s Medical Center Dallas, Dallas, TX 75247 USA; 5 Department of Pediatrics, University of Texas Southwestern Medical Center, Dallas, TX 75390 USA; 6 Department of Neurology, University of Texas Southwestern Medical Center, Dallas, TX 75390 USA; 7 Department of Psychiatry, University of Texas Southwestern Medical Center, Dallas, TX 75390 USA; 8 Department of Neuroscience, University of Texas Southwestern Medical Center, Dallas, TX 75390 USA; 9 Center for the Genetics of Host Defense, University of Texas Southwestern Medical Center, Dallas, TX 75390 USA; 10 Peter O’Donnell Jr. Brain Institute, University of Texas Southwestern Medical Center, Dallas, TX 75390 USA

**Keywords:** Autism spectrum disorders, Medical genomics

## Abstract

Autism spectrum disorder (ASD) comprises neurodevelopmental disorders with wide variability in genetic causes and phenotypes, making it challenging to pinpoint causal genes. We performed whole exome sequencing on a modest, ancestrally diverse cohort of 195 families, including 754 individuals (222 with ASD), and identified 38,834 novel private variants. In 68 individuals with ASD (~30%), we identified 92 potentially pathogenic variants in 73 known genes, including *BCORL1*, *CDKL5*, *CHAMP1*, *KAT6A*, *MECP2*, and *SETD1B*. Additionally, we identified 158 potentially pathogenic variants in 120 candidate genes, including *DLG3*, *GABRQ*, *KALRN*, *KCTD16*, and *SLC8A3*. We also found 34 copy number variants in 31 individuals overlapping known ASD loci. Our work expands the catalog of ASD genetics by identifying hundreds of variants across diverse ancestral backgrounds, highlighting convergence on nervous system development and signal transduction. These findings provide insights into the genetic underpinnings of ASD and inform molecular diagnosis and potential therapeutic targets.

## Introduction

Autism spectrum disorder (ASD) is a collection of neurodevelopmental disorders manifested by impaired social communication, repetitive behaviors, and restricted interests^1^. In addition to these primary symptoms, individuals with ASD often experience comorbidities like intellectual disability, anxiety, depression, attention disorders, and epilepsy^2^. About 1 in 36 children has been identified with ASD according to the latest estimates from CDC’s Autism and Developmental Disabilities Monitoring (ADDM) Network^3^.

ASD etiology includes a substantial genetic component, with a large population-based study including 2 million individuals suggesting that approximately 80% of the variation in the phenotype is attributable to genetic factors^4^. Recent genetic analyses have uncovered that rare variations disrupting gene function, identified through whole exome and whole genome sequencing, have large effect sizes on the disorder^5–7^. However, the genetic variants identified to date only account for a small fraction of the overall disease burden^8^, and each of the currently known ASD genes accounts for less than ~2% of cases^9^. Although hundreds of ASD susceptibility genes have been identified, research suggests that there may be 400–1000 genes associated with ASD susceptibility^10,11^. Thus, fully understanding the genetic architecture of ASD will require continuous efforts to sequence samples from ASD cohorts. Importantly, the majority of studies are focused on single ancestries—most frequently European ancestry—which limits genetic discovery, introduces bias, and misses ancestry-specific effects, reducing generalizability.

We enrolled a modest familial ASD cohort from diverse ancestral backgrounds and performed whole exome sequencing (WES) on a total of 754 individuals from 195 families, including 222 probands with ASD and their family members without ASD. We focused on spontaneous and inherited rare deleterious variants as pathogenic candidates. In total, we identified 92 potentially pathogenic variants in 73 genes that have been previously implicated in ASD or other neurodevelopmental disorders, and 158 potentially pathogenic coding variants in 120 candidate ASD genes. We also identified 34 copy number variants (CNVs) in all individuals with ASD that overlap with known loci. Through this study in a multi-ancestral ASD cohort, we identified potentially pathogenic variants in known ASD or neurodevelopmental disease genes enriched for nervous system development and neurogenesis and novel genes enriched for regulation of signal transduction. Our study underscores the significance of genetic diversity in ASD research and highlights the roles of the identified genes in brain development.

## Results

### Clinical characteristics of the ASD cohort

**Fig. 1:**
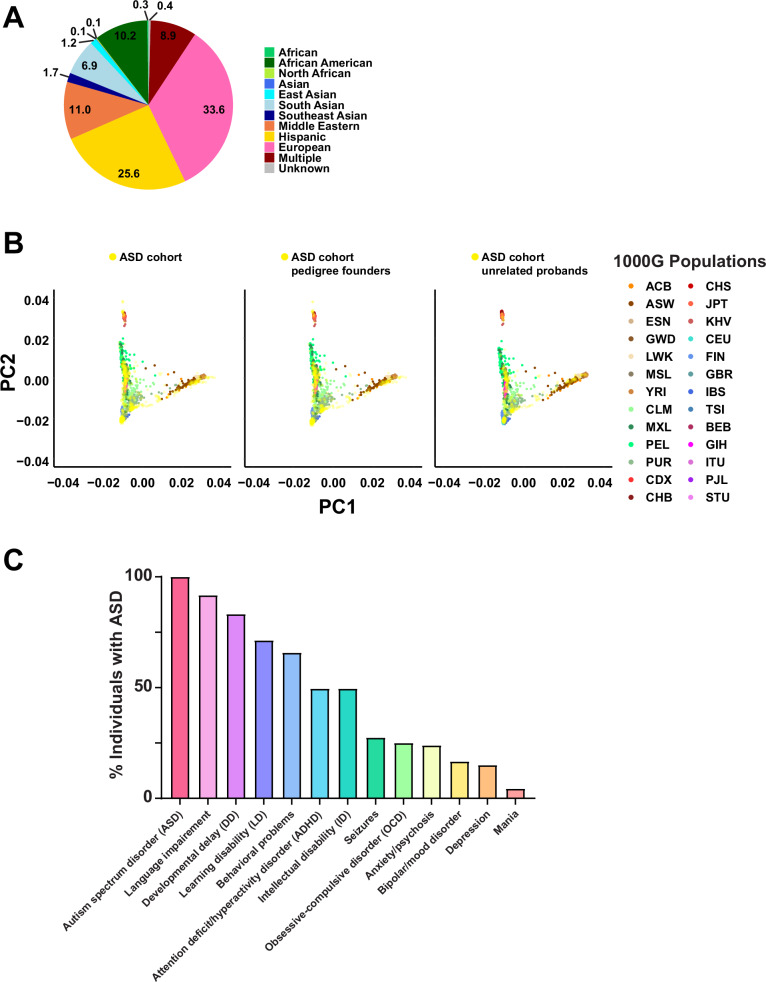
Ancestral diversity and phenotypic spectrum of the ASD cohort. **A** Pie chart depicting the ancestral diversity of the ASD cohort. Multiple refers to individuals with multiple ancestries. **B** Principal component analysis (PCA) of the ASD cohort samples combined with the 1000G populations, using the entire ASD cohort (left), the pedigree founders (middle), or the unrelated probands (right). The ASD cohort is represented in yellow. The 1000G populations are: ACB African Caribbeans in Barbados, ASW Americans of African Ancestry in Southwest USA, ESN Esan in Nigeria, GWD Gambian in Western Divisions in Gambia, LWK Luhya in Webuye, Kenya, MSL Mende in Sierra Leone, YRI Yoruba in Ibadan, Nigeria, CLM Colombians from Medellin, Colombia, MXL Mexican Ancestry from Los Angeles, USA, PEL Peruvians from Lima, Peru, PUR Puerto Ricans from Puerto Rico, CDX Chinese Dai in Xishuangbanna, China, CHB Han Chinese in Beijing, China, CHS Southern Han Chinese, JPT Japanese in Tokyo, Japan, KHV Kinh in Ho Chi Minh City, Vietnam, CEU Utah Residents (CEPH) with Northern and Western European Ancestry, FIN Finnish in Finland, GBR British in England and Scotland, IBS Iberian Population in Spain, TSI Toscani in Italia, BEB Bengali from Bangladesh, GIH Gujarati Indian from Houston, Texas, ITU Indian Telugu from the UK, PJL Punjabi from Lahore, Pakistan, STU Sri Lankan Tamil from the UK. Population abbreviations are also defined in Supplementary Data 11. **C** The prevalence of neurodevelopmental and neuropsychiatric conditions in the ASD cohort. ASD was diagnosed in all 222 probands (100%). Language impairment was the most commonly reported phenotype (91.72%).

A total of 195 simplex and multiplex families who have at least one child diagnosed with ASD were enrolled in our study (Supplementary Data 1). The enrolled families represent diverse ancestral backgrounds, including African American, Asian, Hispanic, Middle Eastern, Native American, and European (Fig. 1A). We used principal component analysis (PCA) to explore the ancestry of the families in the cohort (Fig. 1B). Our cohort clustered across the different subpopulations of the 1000 Genomes project (1000G)^12^. Given that our cohort does not comprise a specific population, this finding is consistent with expectations. The cohort included a total of 222 individuals with ASD and their family members without ASD (165 fathers, 188 mothers, 5 grandmothers, and 174 siblings), and we observed a male-to-female ratio of 2.7:1 (162 males, 60 females) among individuals with ASD. This is slightly lower than the more recent estimates of ~3:1^13,14^ or previous estimates of ~4:1^13^. Parental age, which is a possible risk factor for ASD^15^, was not significantly different at the time of birth of individuals with ASD compared to offspring with no ASD (Supplementary Fig. 1). A standardized medical questionnaire was collected from each of the 195 participating families and reviewed along with available medical records for the presence of clinical comorbidities commonly associated with ASD and other neurodevelopmental disorders, including attention deficit/hyperactivity disorder (ADHD), language delay or impairment, cognitive impairment including intellectual disability, specific learning disability, aggression or challenging behaviors, mood disorders (i.e., anxiety, depression, obsessive-compulsive disorder (OCD), bipolar disorder), seizures, and sleep problems. There were 222 individuals diagnosed with ASD and 532 participants without ASD. Of those individuals with ASD where complete information for a specific phenotype was available, 91.72% had language impairment, 83.21% had developmental delay, 71.31% had learning disability, 65.81% had behavioral problems, 49.55% had ADHD, 49.54% had intellectual disability, 27.45% had seizures, and 25% had OCD (Fig. 1C). Other medical comorbidities were seen at lower frequencies, including environmental and food allergies, and respiratory, gastrointestinal, and vision problems. Demographics and clinical information for the cohort are provided in Fig. 1, Table 1 and Supplementary Data 1.

**Table 1:** Demographics and clinical information for the ASD cohort

A. Demographics			
Number of individuals	All	Males	Females
Cohort (*N*)	754	411	343
Parents (*N*)	353	165	188
Age (mean, years)	44.1	45.3	43.0
Age (median, years)	44	44	43
Non-ASD siblings (*N*)	174	82	92
Age (mean, years)	15.8	15.3	16.2
Age (median, years)	15	13.5	16
Paternal age at birth (mean, years)	31.5	31.4	31.5
Maternal age at birth (mean, years)	28.9	29.6	28.4
Individuals with ASD (*N*)	222	162	60
Age (mean, years)	14.5	14.3	15.1
Age (median, years)	13	12	14
Paternal age at birth (mean, years)	32.4	32.2	33.0
Maternal age at birth (mean, years)	30.0	30.2	29.4

B. Ancestry		
Ancestry	Number of individuals	% of individuals
African	2	0.3
African American	77	10.2
North African	1	0.1
Asian	1	0.1
East Asian	9	1.2
South Asian	52	6.9
Southeast Asian	13	1.7
Middle Eastern	83	11.0
Hispanic	193	25.6
European	253	33.6
Multiple	67	8.9
Unknown	3	0.4

C. Clinical information			
Clinical symptoms	Number of individuals tested	Number of individuals with phenotype	% of individuals with ASD
Autism spectrum disorder (ASD)	222	222	100.00
Language impairment	145	133	91.72
Developmental delay (DD)	137	114	83.21
Learning disability (LD)	122	87	71.31
Behavioral problems	117	77	65.81
Attention deficit/hyperactivity disorder (ADHD)	111	55	49.55
Intellectual disability (ID)	109	54	49.54
Seizures	102	28	27.45
Obsessive-compulsive disorder (OCD)	96	24	25.00
Anxiety/psychosis	92	22	23.91
Bipolar/mood disorder	90	15	16.66
Depression	93	14	15.05
Mania	91	4	4.40

Age refers to current age in 2024. Multiple refers to individuals with multiple ancestries.

*ASD* autism spectrum disorder, *DD* developmental delay, *LD* learning disability, *ADHD* attention deficit/hyperactivity disorder, *ID* intellectual disability, *OCD* obsessive-compulsive disorder.

### Whole exome sequencing and variant discovery in the ASD cohort

**Fig. 2:**
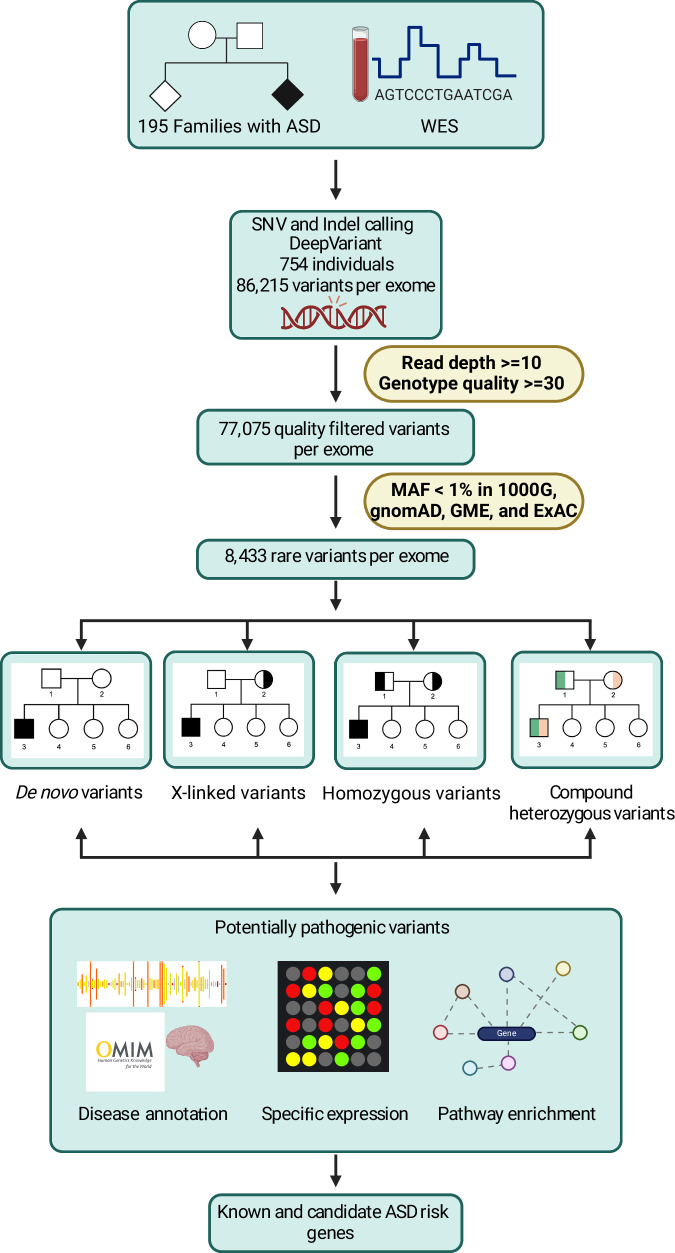
Overview diagram of study analyses. Whole exome sequencing (WES) was performed on 754 individuals from 195 families, including 222 probands with ASD and their family members without ASD (165 fathers, 188 mothers, 5 grandmothers, and 174 siblings). Single nucleotide variants (SNVs) and small insertions or deletions (indels) were called using DeepVariant. Variant quality filtering was performed as described in the Materials and Methods. Rare de novo or inherited (X-linked, homozygous, and compound heterozygous) variants were annotated to identify potentially pathogenic variants. Risk genes were prioritized by disease annotation, specific expression, and pathway enrichment. MAF minor allele frequency. This figure was created with BioRender.com.

We performed WES on samples from 754 individuals, including 222 individuals with ASD. The average read depth was 46X, with no differences in depth of sequencing with respect to phenotypic status, sex, or family relationships (Supplementary Fig. 2A–C). On average, 99.29% and 93.9% of bases were covered at a mean read depth of at least 10X and 20X, respectively (Supplementary Fig. 2D). An average of 86,215 total variants were identified per exome, of which an average of 73,132 were single nucleotide variants (SNVs) and 13,083 were insertions or deletions (indels) (Supplementary Data 2). After applying read depth and quality filters, 77,075 variants per exome remained, of which an average of 65,907 were SNVs and 11,168 were indels (Supplementary Data 2). A detailed summary of our WES data processing and variant filtration pipeline is shown in Fig. 2. We filtered for rare variants with a minor allele frequency (MAF) < 1% in all annotated population databases ((1000G)^12^, Genome Aggregation Database (gnomAD)^16,17^, the Greater Middle East Variome project (GME)^18^, and The Exome Aggregation Consortium (ExAC)^19^), identifying on average 8433 rare variants per exome, of which 7002 were heterozygous and 1431 were homozygous (Supplementary Data 2). We defined potentially damaging variants as the subset of rare exonic or splice site (referred to as coding) variants that are also predicted to be damaging by at least 1 of the 2 algorithms used: SIFT and PolyPhen-2 HumVar. There was no significant difference in the number of potentially damaging variants between sexes for individuals with ASD in the cohort (Supplementary Fig. 3). To assess for an excess of potentially damaging variants in individuals with ASD compared to individuals without ASD, we performed a burden analysis. We found no difference between individuals with or without ASD in the burden of rare variants with total coding, nondisrupting, missense damaging, or loss of function effects (Supplementary Fig. 4). This outcome is expected, given our modest sample size and the fact that ASD comprises individually rare diseases with genetic heterogeneity, caused by rare alleles of substantial impact. Therefore, observing an excess of these variations requires studying much larger cohorts capable of capturing this heterogeneity. We discovered an average of 5959 novel variants per exome that have not been reported in any of the populations in the public databases that we used for annotation (Supplementary Data 2). Furthermore, we found an average of 52 novel variants per individual that were private (71 for parents, 34 for offspring), meaning they have not been reported in any of the annotated populations and they were not present in any other individual in the cohort (Supplementary Data 3). In total, there were 38,834 novel private variants across all individuals in the cohort (Supplementary Data 3). As expected, more private variants were present in parents compared with offspring (Supplementary Fig. 5). We identified an average of 15 (20 for parents, 9 for offspring) private coding variants per exome, of which an average of 6 (8 for parents, 4 for offspring) per exome were nonsynonymous and predicted to be potentially damaging by at least 1 of the 2 algorithms used, SIFT and PolyPhen-2 HumVar (Supplementary Data 3).

### Identification of candidate ASD variants

**Fig. 3:**
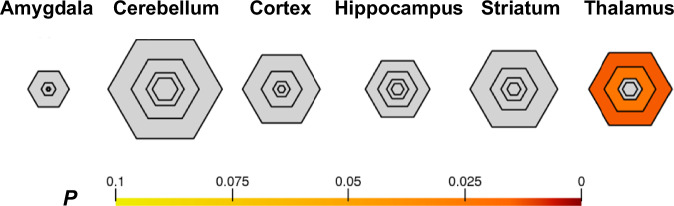
Enrichment of the identified ASD genes in the thalamus. Bullseye plot of specific expression analysis (SEA) of genes harboring the prioritized variants across brain regions and development. SEA revealed that genes with possibly damaging variants detected in the ASD cohort were enriched during young adulthood in the thalamus. The color bar shows Benjamini–Hochberg corrected *p*.

For candidate ASD variant discovery, we initially focused on rare nonsynonymous exonic or splice site variants that were either de novo or segregated with ASD in the family under homozygous, compound heterozygous, or X-linked inheritance. We identified an average of 4 de novo variants (2 coding) per offspring with ASD (Supplementary Data 4). In addition, we identified an average of 155 inherited homozygous variants (38 coding) and 10 compound heterozygous variants in 3 genes per offspring with ASD (Supplementary Data 4). We also identified an average of 16 recessive X-linked variants in male offspring with ASD (8 coding) (Supplementary Data 4). We did not find a significant correlation between the number of de novo variants and maternal or paternal age at birth of an offspring with ASD (Supplementary Fig. 6). In total, we identified 630 genes harboring 1503 rare nonsynonymous exonic or splice site variants that are predicted to be potentially damaging by at least 1 of the 2 algorithms used, SIFT and PolyPhen-2 HumVar (Supplementary Data 5). The shared symptoms among individuals with ASD suggest the existence of a functional convergence downstream of loci that contribute to the condition. To investigate if there is selective expression of at least some of these 630 genes in different brain regions, we conducted specific expression analysis (SEA) using human transcriptomics data from the BrainSpan collection^20^. We found that genes with variants detected in the individuals with ASD in our cohort were enriched in the thalamus (*p* = 0.014) (Fig. 3 and Supplementary Data 6), including *AR*, *ATP1A3*, *SCN1A*, and *SLC7A3*.

### Variants in known ASD or neurodevelopmental disease genes

Table 2 summarizes the potentially pathogenic variants in 73 known ASD or neurodevelopmental disease genes for each individual with ASD after variant prioritization. Out of these genes, 40 are reported in the Simons Foundation Autism Research Initiative (SFARI) Gene database^21^, and the rest are OMIM-annotated disease genes associated with relevant phenotypes, including neurodevelopmental disorder, intellectual disability, developmental delay, and epilepsy. These genes were significantly enriched in pathways involving nervous system development, neurogenesis, and neuronal differentiation (Supplementary Data 7). We identified 92 unique variants in 68 individuals with ASD (~1–3 per individual). Twenty-six individuals with ASD had coding variants in 19 syndromic ASD genes: *CDKL5* (3 probands), *DMD* (3 probands), *BCORL1* (2 probands), and *SETD1B* (2 probands). *ARID1B*, *ATP1A3*, *CHAMP1*, *CNOT1*, *FRMPD4*, *HUWE1*, *KAT6A*, *KMT2C*, *MECP2*, *PACS2*, *PHF21A*, *SCN1A*, *SLC6A1*, *SMARCA2*, *TFE3*, and *ZMYM3* are other syndromic ASD genes harboring variants in single probands. Twenty-three individuals with ASD had coding variants in 21 nonsyndromic ASD genes having a SFARI Gene^21^ score of 1 or 2: *NEXMIF* (2 probands) and *NLGN4X* (2 probands). *AR*, *ARHGEF10*, *ASTN2*, *AUTS2*, *BIRC6*, *CACNA1F*, *DLG4*, *DYNC1H1*, *IL1RAPL1*, *ITPR1*, *OPHN1*, *PCDHA5*, *SKI*, *SLC7A3*, *SYN1*, *TOP2B*, *WNK3*, *YEATS2*, and *ZC3H4* are other ASD genes harboring variants in single probands. Thirty-two probands had other coding variants in 33 neurodevelopmental disease genes, with 2 genes—*ADGRV1* and *ATP7A*—having variants in 2 probands each. *ACSL4*, *ARHGAP31*, *ARMC9*, *ATP2B3*, *ATP6AP2*, *BCAP31*, *CCDC22*, *CHD5*, *DBR1*, *DCTN1*, *DHX37*, *FGD1*, *HDAC6*, *IGBP1*, *KIF1C*, *MINPP1*, *MPDZ*, *NOTCH1*, *NRG1*, *OBSL1*, *PIGG*, *PLXNA1*, *SAMD9L*, *SCN3A*, *SLC13A3*, *SRPX2*, *TMEM151A*, *TNRC6A*, *TRIM71*, *TRNT1*, and *ZNF148* are other neurodevelopmental disease genes harboring variants in single probands. Three probands had coding variants in two neurodevelopmental genes each: MC-159-5 (*ADGRV1* and *KIF1C*), MC-161-3 (*MPDZ* and *NRG1*), and MC-172-3 (*OBSL1* and *SAMD9L*).

**Table 2:** Potentially pathogenic variants in known ASD and neurological disease genes identified in individuals with ASD from the cohort

Individual with ASD	Inheritance	Variant(s)	Variant type	Gene	Mutation	Relevant OMIM Phenotype	SFARI score	pLI score	LOEUF score	*Z* score
JC_19_3	De novo	chrX:154030912-154030912:G:A	nonsynonymous SNV	*MECP2*	p.R318C	Rett syndrome, Encephalopathy, Intellectual developmental disorder	1S	0.89382	0.407	2.893
JC_20_3	X-Linked	chrX:32389610-32389610:C:T	nonsynonymous SNV	*DMD*	p.R129H	–	S	1	0.154	10.694
JC_22_4	De novo	chr3:125233501-125233501:G:A	stopgain	*ZNF148*	p.Q409X	Global developmental delay, absent or hypoplastic corpus callosum	–	0.99997	0.103	4.9945
JC_24_3	Inherited homozygous	chr12:124952446-124952446:C:G	nonsynonymous SNV	*DHX37*	p.R940S	Neurodevelopmental disorder	–	0.99252	0.289	5.8911
JC_25_3	De novo	chr3:4733157-4733157:A:T	nonsynonymous SNV	*ITPR1*	p.S1701C	Gillespie syndrome, Spinocerebellar ataxia	2	1	0.134	9.9326
JC_27_3	Inherited homozygous	chr16:58543412-58543412:-:A	frameshift insertion	*CNOT1*	p.L1544Sfs*22	Vissers-Bodmer syndrome, Holoprosencephaly	2S	1	0.038	10.279
JC_32_3	De novo	chr3:11025863-11025863:A:G	clinvar AN	*SLC6A1*	p.N136D	Intellectual developmental disorder	1S	0.99993	0.15	5.0491
MC_003_3	De novo	chr1:6142418-6142418:T:C	nonsynonymous SNV	*CHD5*	p.K744R	Parenti-Mignot neurodevelopmental syndrome	–	1	0.157	8.4428
MC_004_3	De novo	chr19:41986183-41986183:C:G	nonsynonymous SNV	*ATP1A3*	p.C146S	Alternating hemiplegia, CAPOS syndrome	2S	1	0.062	6.3973
MC_005_3	X-Linked	chrX:47574709-47574709:G:C	nonsynonymous SNV	*SYN1*	p.Q458E	–	1	0.99216	0.251	3.8157
MC_005_3	X-Linked	chrX:18595364-18595364:A:G	nonsynonymous SNV	*CDKL5*	p.H254R	Developmental and epileptic encephalopathy	1S	0.99932	0.226	4.9513
MC_014_3	Inherited homozygous	chr4:539215-539215:C:T	nonsynonymous SNV	*PIGG*	p.T800M	Neurodevelopmental disorder	–	5.4258E−15	0.988	1.615
MC_017_3	De novo	chr2:165991869-165991872:CTCA:-	frameshift deletion	*SCN1A*	p.S1801Rfs*56	Developmental and epileptic encephalopathy, Dravet syndrome	1S	1	0.071	8.5198
MC_019_3^a^	De novo	chr3:32890881-32890881:G:C	nonsynonymous SNV	*TRIM71*	p.S1801Rfs*56	Hydrocephalus	–	0.99969	0.172	4.6883
MC_022_3^a^	X-Linked	chrX:70134643-70134643:G:T	nonsynonymous SNV	*IGBP1*	p.Q103H	Impaired intellectual development	–	0.98274	0.242	3.2578
MC_024_3	Inherited homozygous	chr3:25664257-25664257:C:G	nonsynonymous SNV	*TOP2B*	p.G14A	–	2	0.99989	0.247	6.9742
MC_024_3	X-Linked	chrX:78014707-78014707:A:G	nonsynonymous SNV	*ATP7A*	p.T740A	Occipital horn syndrome, Menkes disease	–	0.99983	0.216	5.468
MC_025_3	X-Linked	chrX:130016202-130016202:C:T	nonsynonymous SNV	*BCORL1*	p.H1144Y	Shukla-Vernon syndrome	S	0.99999	0.152	5.6731
MC_025_4	X-Linked	chrX:130016202-130016202:C:T	nonsynonymous SNV	*BCORL1*	p.H1144Y	Shukla-Vernon syndrome	S	0.99999	0.152	5.6731
MC_027_3	X-Linked	chrX:29955423-29955423:A:C	nonsynonymous SNV	*IL1RAPL1*	p.E565A	Intellectual developmental disorder	2	0.99886	0.197	4.3584
MC_027_3	X-Linked	chrX:49248232-49248232:G:C	nonsynonymous SNV	*CCDC22*	p.E378D	Ritscher-Schinzel syndrome	–	0.99979	0.123	4.5588
MC_028_3	X-Linked	chrX:49222958-49222958:A:C	nonsynonymous SNV	*CACNA1F*	p.F686V	–	2	1.2337E−05	0.448	5.4046
MC_032_3	Inherited homozygous	chr2:165090971-165090971:G:A	nonsynonymous SNV	*SCN3A*	p.P1679S	Developmental and epileptic encephalopathy	–	1	0.174	7.6338
MC_042_3	Compound heterozygous	chr14:101988835-101988835:G:A	nonsynonymous SNV	*DYNC1H1*	p.G951R	–	1	1	0.08	13.319
MC_042_3	Compound heterozygous	chr14:102018473-102018473:G:A	nonsynonymous SNV	*DYNC1H1*	p.V2734M	–	1	1	0.08	13.319
MC_044_3	X-Linked	chrX:153723526-153723526:G:A	nonsynonymous SNV	*BCAP31*	p.H47Y	Cerebral hypomyelination	–	0.43366	0.65	2.2884
MC_045_3	X-Linked	chrX:153556212-153556212:G:A	nonsynonymous SNV	*ATP2B3*	p.R741H	Spinocerebellar ataxia	–	0.99945	0.222	4.9998
MC_053_3	Inherited homozygous	chr20:46613719-46613719:G:C	nonsynonymous SNV	*SLC13A3*	p.R40G	Leukoencephalopathy	–	9.4522E−07	0.834	2.2133
MC_060_3	X-Linked	chrX:71249094-71249094:G:A	nonsynonymous SNV	*ZMYM3*	p.R516C	Intellectual developmental disorder	S	1	0.106	6.0468
MC_063_4	Compound heterozygous	chr3:119414252-119414252:G:A	nonsynonymous SNV	*ARHGAP31*	p.G775S	Adams-Oliver syndrome	–	0.99999	0.192	6.2345
MC_063_4	Compound heterozygous	chr3:119415525-119415525:C:T	nonsynonymous SNV	*ARHGAP31*	p.A1199V	Adams-Oliver syndrome	–	0.99999	0.192	6.2345
MC_064_3	X-Linked	chrX:18625233-18625233:G:C	nonsynonymous SNV	*CDKL5*	p.D828H	–	1S	0.99932	0.226	4.9513
MC_069_3^a^	De novo	chr11:66295148-66295148:G:A	nonsynonymous SNV	*TMEM151A*	p.R301H	Episodic kinesigenic dyskinesia	–	0.0029247	0.943	1.7136
MC_070_5	X-Linked	chrX:74743416-74743416:C:T	nonsynonymous SNV	*NEXMIF*	p.D381N	Intellectual developmental disorder	1	–	–	–
MC_073_3	Compound heterozygous	chr3:3147641-3147641:G:C	nonsynonymous SNV	*TRNT1*	p.D312H	Developmental delay	–	0.00015886	0.876	1.9533
MC_073_3	Compound heterozygous	chr3:3148141-3148141:-:A	frameshift insertion	*TRNT1*	p.K413Efs*34	Developmental delay	–	0.00015886	0.876	1.9533
MC_081_4	X-Linked	chrX:74742674-74742674:C:T	nonsynonymous SNV	*NEXMIF*	p.R628Q	Intellectual developmental disorder	1	–	–	–
MC_088_4	Compound heterozygous	chr2:32467973-32467973:A:G	nonsynonymous SNV	*BIRC6*	p.H1881R	–	2	1	0.104	12.544
MC_088_4	Compound heterozygous	chr2:32597936-32597936:C:T	nonsynonymous SNV	*BIRC6*	p.R4600C	–	2	1	0.104	12.544
MC_099_3^a^	De novo	chr17:7218589-7218589:G:T	nonsynonymous SNV	*DLG4*	p.P24T	Intellectual developmental disorder	1	0.99954	0.238	5.4593
MC_102_3^a^	De novo	chr1:2229037-2229037:T:G	nonsynonymous SNV	*SKI*	p.F91V	Shprintzen-Goldberg syndrome	1	0.99901	0.194	4.3963
MC_103_3	Compound heterozygous	chr9:116425970-116425970:C:T	nonsynonymous SNV	*ASTN2*	p.E353K	–	2	0.99971	0.246	6.1231
MC_103_3	Compound heterozygous	chr9:116426065-116426065:C:T	nonsynonymous SNV	*ASTN2*	p.R321Q	–	2	0.99971	0.246	6.1231
MC_110_3	X-Linked	chrX:54250099-54250099:G:A	nonsynonymous SNV	*WNK3*	p.R870W	–	2	0.99999	0.191	6.2565
MC_111_3	De novo	chr7:70766248-70766248:C:T	nonsynonymous SNV	*AUTS2*	p.H535Y	Intellectual developmental disorder	1	0.99934	0.253	5.7821
MC_112_3^a^	De novo	chr8:41933512-41933512:C:T	nonsynonymous SNV	*KAT6A*	p.D1570N	Arboleda-Tham syndrome	2S	1	0.069	8.6737
MC_116_3	De novo	chr2:74370632-74370632:A:T	nonsynonymous SNV	*DCTN1*	p.I212N	Neuronopathy, Perry syndrome	–	0.084251	0.364	5.8791
MC_117_3	X-Linked	chrX:31121880-31121880:T:A	nonsynonymous SNV	*DMD*	p.M608L	–	S	1	0.154	10.694
MC_117_4	X-Linked	chrX:31121880-31121880:T:A	nonsynonymous SNV	*DMD*	p.M608L	–	S	1	0.154	10.694
MC_118_3	X-Linked	chrX:68053801-68053801:T:C	nonsynonymous SNV	*OPHN1*	p.D723G	Intellectual developmental disorder	2	0.99985	0.161	4.8611
MC_120_3	Compound heterozygous	chr6:156778045-156778045:C:T	nonsynonymous SNV	*ARID1B*	p.S39F	Intellectual developmental disorder	1S	1	0.102	8.4054
MC_120_3	Compound heterozygous	chr6:157201357-157201357:C:G	nonsynonymous SNV	*ARID1B*	p.P878R	Intellectual developmental disorder	1S	1	0.102	8.4054
MC_120_3	X-Linked	chrX:48814732-48814732:G:A	nonsynonymous SNV	*HDAC6*	p.A331T	Hydrocephaly	–	1	0.072	5.9451
MC_124_6	De novo	chr13:114324638-114324638:G:T	stopgain	*CHAMP1*	p.E266X	Neurodevelopmental disorder	1S	0.99197	0.271	4.0836
MC_126_3	X-Linked	chrX:54455715-54455715:G:A	nonsynonymous SNV	*FGD1*	p.R638C	Intellectual developmental disorder	–	0.9997	0.196	4.9187
MC_134_3^a^	De novo	chr5:140823339-140823339:C:A	nonsynonymous SNV	*PCDHA5*	p.L522M	–	2	5.8373E−08	0.879	2.0539
MC_136_3	X-Linked	chrX:70928613-70928613:T:C	nonsynonymous SNV	*SLC7A3*	p.S184G	–	2	0.99614	0.182	3.7525
MC_138_3	X-Linked	chrX:5893394-5893394:G:A	nonsynonymous SNV	*NLGN4X*	p.T625I	Intellectual developmental disorder	1	0.99267	0.249	3.8359
MC_138_4	X-Linked	chrX:5893394-5893394:G:A	nonsynonymous SNV	*NLGN4X*	p.T625I	Intellectual developmental disorder	1	0.99267	0.249	3.8359
MC_140_3	De novo	chr16:24776966-24776966:C:A	nonsynonymous SNV	*TNRC6A*	p.P66H	Epilepsy	–	1	0.159	8.3756
MC_146_3	Compound heterozygous	chr9:136509800-136509800:T:C	nonsynonymous SNV	*NOTCH1*	p.T968A	Adams-Oliver syndrome	–	1	0.097	9.1999
MC_146_3	Compound heterozygous	chr9:136522960-136522960:G:A	nonsynonymous SNV	*NOTCH1*	p.T211I	Adams-Oliver syndrome	–	1	0.097	9.1999
MC_146_3	De novo	chr12:121806064-121806064:C:-	frameshift deletion	*SETD1B*	p.V169Sfs*46	Intellectual developmental disorder	2S	1	0.151	6.7395
MC_148_3	De novo	chr14:105381945-105381945:C:T	nonsynonymous SNV	*PACS2*	p.R434W	Developmental and epileptic encephalopathy	S	0.99583	0.279	5.4113
MC_148_3	X-Linked	chrX:67546365-67546365:C:G	nonsynonymous SNV	*AR*	p.R407G	Neuropathy	2	0.98837	0.291	4.2459
MC_154_3^a^	X-Linked	chrX:109674452-109674452:T:C	nonsynonymous SNV	*ACSL4*	p.S359G	Intellectual developmental disorder	–	0.98103	0.306	4.1113
MC_154_3^a^	X-Linked	chrX:18604102-18604102:C:T	nonsynonymous SNV	*CDKL5*	p.T393I	Developmental and epileptic encephalopathy	1S	0.99932	0.226	4.9513
MC_155_3	X-Linked	chrX:77989260-77989260:T:C	nonsynonymous SNV	*ATP7A*	p.I213T	Occipital horn syndrome, Menkes disease	–	0.99983	0.216	5.468
MC_156_3^a^	De novo	chr2:231270991-231270991:C:-	frameshift deletion	*ARMC9*	p.L344Ffs*46	Joubert syndrome	–	6.2032E−17	1.053	1.2891
MC_158_3^a^	De novo	chr19:47081616-47081616:A:G	nonsynonymous SNV	*ZC3H4*	p.F446S	–	2	1	0.054	6.8501
MC_158_3^a^	De novo	chr9:2056699-2056699:C:T	nonsynonymous SNV	*SMARCA2*	p.R401C	Nicolaides-Baraitser syndrome	1S	1	0.203	7.6947
MC_159_3	Inherited homozygous	chr5:90694224-90694224:G:A	nonsynonymous SNV	*ADGRV1*	p.A2490T	Usher syndrome	–	–	–	–
MC_159_5	De novo	chrX:49038391-49038391:G:C	nonsynonymous SNV	*TFE3*	p.R91G	Intellectual developmental disorder	S	0.97985	0.29	3.5174
MC_159_5	Inherited homozygous	chr17:5004866-5004866:G:A	nonsynonymous SNV	*KIF1C*	p.R344H	Spastic ataxia	–	0.71767	0.341	5.5201
MC_159_5	Inherited homozygous	chr5:90694224-90694224:G:A	nonsynonymous SNV	*ADGRV1*	p.A2490T	Usher syndrome	–	–	–	–
MC_160_3	De novo	chr3:183715186-183715186:-:A	frameshift insertion	*YEATS2*	p.E10Rfs*5	Epilepsy	2	0.99639	0.28	6.5648
MC_160_3	De novo	chr3:127012021-127012021:A:T	nonsynonymous SNV	*PLXNA1*	p.T726S	Dworschak-Punetha neurodevelopmental syndrome	–	0.99951	0.262	7.2148
MC_161_3^a^	De novo	chr9:13192219-13192219:G:T	nonsynonymous SNV	*MPDZ*	p.T627K	Hydrocephalus	–	5.8009E−38	0.89	2.4713
MC_161_3^a^	De novo	chr8:32763319-32763319:A:T	nonsynonymous SNV	*NRG1*	p.H303L	Schizophrenia	–	0.99665	0.258	4.5687
MC_162_3^a^	De novo	chr8:1898505-1898505:C:G	nonsynonymous SNV	*ARHGEF10*	p.Q506E	–	2	6.7739E−30	0.976	1.7165
MC_163_3	Compound heterozygous	chr7:152145253-152145253:C:T	nonsynonymous SNV	*KMT2C*	p.G4692S	Kleefstra syndrome	1S	1	0.122	12.592
MC_163_3	Compound heterozygous	chr7:152311917-152311917:G:C	nonsynonymous SNV	*KMT2C*	p.S207C	Kleefstra syndrome	1S	1	0.122	12.592
MC_166_3^a^	De novo	chr3:138163793-138163793:A:T	nonsynonymous SNV	*DBR1*	p.H260Q	Encephalitis	–	1.1785E−08	1.016	1.4986
MC_166_3^a^	De novo	chr12:121814182-121814182:G:T	nonsynonymous SNV	*SETD1B*	p.C656F	Intellectual developmental disorder	2S	1	0.151	6.7395
MC_170_3	X-Linked	chrX:100665351-100665351:C:T	nonsynonymous SNV	*SRPX2*	p.A214V	Rolandic epilepsy, impaired intellectual development	–	0.04812	0.538	3.2685
MC_171_3	De novo	chr10:87505073-87505073:A:G	nonsynonymous SNV	*MINPP1*	p.Y53C	Pontocerebellar hypoplasia	–	0.00045482	0.76	2.3797
MC_171_3	X-Linked	chrX:32463545-32463545:T:A	nonsynonymous SNV	*DMD*	p.N1101I	–	S	1	0.154	10.694
MC_172_3^a^	De novo	chr2:219562001-219562001:G:C	stopgain	*OBSL1*	p.Y987X	3-M syndrome	–	9.9902E−26	0.878	2.4208
MC_172_3^a^	De novo	chr7:93134087-93134087:G:A	nonsynonymous SNV	*SAMD9L*	p.R629W	Ataxia-pancytopenia syndrome, Spinocerebellar ataxia	–	5.5651E−15	0.783	2.8638
MC_173_3	X-Linked	chrX:12716341-12716341:C:T	nonsynonymous SNV	*FRMPD4*	p.R588W	Intellectual developmental disorder	S	1	0.083	5.536
MC_174_3	X-Linked	chrX:40599599-40599599:G:A	nonsynonymous SNV	*ATP6AP2*	p.R199H	Intellectual developmental disorder	–	0.87089	0.429	2.8047
MC_174_3	X-Linked	chrX:53583851-53583851:C:T	nonsynonymous SNV	*HUWE1*	p.G1743R	Intellectual developmental disorder	S	1	0.060	11.175

All variants are exonic. For SFARI score, S denotes syndromic genes. LOEUF loss-of-function observed/expected upper bound fraction.

^a^Samples with a missing parent sample where compound heterozygous variant calling was not possible and de novo, inherited homozygous, and X-linked variant calling relied on one parent only.

### Variants in new candidate ASD genes

We identified 158 potentially pathogenic coding variants in 120 candidate ASD genes after variant prioritization (Table 3). Gene ontology analysis revealed that several of the candidate ASD genes are involved in signal transduction and synaptic activity such as *DLG3*, *GABRQ*, *KALRN*, *KCTD16*, *P2RX4*, *PKP4*, *SLC8A3*, and *TENM2* (Supplementary Data 7). Multiple variants were observed in candidate genes: *ATG4A*, *CNGA2*, *CROCC*, *FAM47C*, *FRMPD3*, *GABRQ*, *GPRASP1*, *MAGEC3*, *MXRA5*, *OR5H1*, *PWWP3B*, *SLITRK4*, *TRPC5*, *TSPYL2*, and *ZNF630*. Since we observed more than one potentially pathogenic variant (in known and/or novel genes) in some probands, we also ranked them according to their likelihood of causing the disease in the proband (Supplementary Data 8). In proband MC-017-3, there were two variants found in *SCN1A* and *RBMX2*. The *SCN1A* variant was prioritized over the *RBMX2* variant as *SCN1A* is a known ASD gene, according to the SFARI Gene database^21^. Similarly, in proband MC-174-3, a variant in *HUWE1*, a known neurodevelopmental disease gene^22,23^, was ranked above a variant in another known neurodevelopmental disease gene *ATP6AP2*^24,25^ based on AlphaMissense scores, and above a variant in the novel gene *MTM1*.

**Table 3:** Potentially pathogenic variants in novel candidate ASD genes identified in individuals with ASD from the cohort

Individual with ASD	Inheritance	Variant	Variant type	Gene	Mutation	pLI score	LOEUF score	*Z* score
JC_17_3	X-Linked	chrX:8795197-8795197:C:A	stopgain	*FAM9A*	p.E238X	3.0977E−12	1.907	−1.2931
JC_18_3	De novo	chr12:14478361-14478361:T:C	nonsynonymous SNV	*ATF7IP*	p.S995P	0.99993	0.213	5.852
JC_18_3	X-Linked	chrX:105949622-105949622:C:G	nonsynonymous SNV	*NRK*	p.C1467W	0.99274	0.289	5.7027
JC_20_3	Compound heterozygous	chr1:16938440-16938440:A:G	nonsynonymous SNV	*CROCC*	p.E444G	1.2656E−24	0.71	3.9469
JC_20_3	Compound heterozygous	chr1:16971564-16971564:C:T	nonsynonymous SNV	*CROCC*	p.R1962C	1.2656E−24	0.71	3.9469
JC_20_3	X-Linked	chrX:152945428-152945428:C:A	nonsynonymous SNV	*ZNF185*	p.S95Y	6.3026E−13	1.139	0.98434
JC_20_3	X-Linked	chrX:51744210-51744210:C:T	nonsynonymous SNV	*GSPT2*	p.P195L	0.98321	0.24	3.2676
JC_21_3	X-Linked	chrX:16786504-16786504:A:G	nonsynonymous SNV	*TXLNG*	p.E6G	0.99845	0.158	4.0223
JC_22_3	X-Linked	chrX:3321790-3321790:T:G	nonsynonymous SNV	*MXRA5*	p.T1299P	0.043013	0.398	5.1219
JC_22_4	Inherited homozygous	chr14:21670348-21670348:-:GT	frameshift insertion	*OR4E1*	p.H197Pfs*14	–	–	–
JC_22_4	X-Linked	chrX:3321790-3321790:T:G	nonsynonymous SNV	*MXRA5*	p.T1299P	0.043013	0.398	5.1219
JC_22_5	X-Linked	chrX:3321790-3321790:T:G	nonsynonymous SNV	*MXRA5*	p.T1299P	0.043013	0.398	5.1219
JC_23_3	X-Linked	chrX:10459690-10459690:T:C	nonsynonymous SNV	*MID1*	p.Q468R	0.97967	0.304	3.8121
JC_24_3	Inherited homozygous	chr12:64875644-64875644:C:A	nonsynonymous SNV	*TBC1D30*	p.S690R	0.88919	0.353	4.4099
JC_24_3	Inherited homozygous	chr12:96324082-96324082:G:A	nonsynonymous SNV	*CDK17*	p.T50M	0.99947	0.222	5.0072
JC_24_3	Inherited homozygous	chr12:124332336-124332336:C:T	nonsynonymous SNV	*NCOR2*	p.R2286Q	1	0.169	8.6249
JC_24_3	X-Linked	chrX:107601697-107601697:T:A	nonsynonymous SNV	*FRMPD3*	p.F1253I	0.00019028	0.476	4.6328
JC_24_3	De novo	chr5:139380289-139380289:G:T	nonsynonymous SNV	*SLC23A1*	p.T189N	0.023504	0.537	3.3723
JC_25_3	Inherited homozygous	chr20:53575768-53575768:C:A	nonsynonymous SNV	*ZNF217*	p.C999F	0.99995	0.147	5.0971
JC_27_3	De novo	chr3:52842931-52842931:-:A	frameshift insertion	*STIMATE;STIMATE-MUSTN1*	p.L217Pfs*10	–	–	–
JC_30_3	Inherited homozygous	chr5:113433762-113433762:G:A	stopgain	*TSSK1B*	p.Q360X	0.00013409	1.711	0.17385
JC_30_3	Inherited homozygous	chr15:90916239-90916239:C:T	stopgain	*MAN2A2*	p.R993X	1.4995E−10	0.619	3.9582
MC_001_3	X-Linked	chrX:141838402-141838402:T:A	stopgain	*MAGEC3*	p.Y29X	7.6566E−16	1.722	−0.8259
MC_001_3	X-Linked	chrX:102657809-102657809:T:C	nonsynonymous SNV	*GPRASP1*	p.M1299T	0.31099	0.416	4.2
MC_001_3	X-Linked	chrX:70492240-70492240:C:T	nonsynonymous SNV	*DLG3*	p.H69Y	0.99999	0.09	5.3454
MC_001_4	X-Linked	chrX:102657809-102657809:T:C	nonsynonymous SNV	*GPRASP1*	p.M1299T	0.31099	0.416	4.2
MC_001_4	X-Linked	chrX:70492240-70492240:C:T	nonsynonymous SNV	*DLG3*	p.H69Y	0.99999	0.09	5.3454
MC_009_2^a^	De novo	chr12:109769169-109769169:A:G	nonsynonymous SNV	*FAM222A*	p.N414D	0.85124	0.446	2.7374
MC_009_2^a^	X-Linked	chrX:143629175-143629175:C:T	nonsynonymous SNV	*SLITRK4*	p.R645H	0.79447	0.422	3.3262
MC_012_3	X-Linked	chrX:106207495-106207495:G:A	nonsynonymous SNV	*PWWP3B*	p.R688Q	–	–	–
MC_014_3	Inherited homozygous	chr15:41857344-41857344:-:T	frameshift insertion	*SPTBN5*	p.A2840Gfs*5	8.222E−118	1.062	0.77177
MC_014_3	Inherited homozygous	chr12:49494879-49494879:A:G	nonsynonymous SNV	*SPATS2*	p.N135D	0.97239	0.319	4.2735
MC_014_3	Inherited homozygous	chr1:183553005-183553005:G:A	nonsynonymous SNV	*SMG7*	p.G1114E	0.99998	0.219	6.6005
MC_014_3	X-Linked	chrX:112811368-112811368:G:A	nonsynonymous SNV	*AMOT*	p.S473L	0.99666	0.266	4.8052
MC_015_3^a^	De novo	chr2:17781754-17781757:AAAG:-	frameshift deletion	*GEN1*	p.E849Lfs*26	3.7299E−15	0.951	1.8067
MC_015_3^a^	De novo	chr1:155765155-155765158:GACC:-	frameshift deletion	*GON4L*	p.G1439Sfs*58	0.9515	0.299	7.1272
MC_015_3^a^	De novo	chr11:92761948-92761948:C:A	nonsynonymous SNV	*FAT3*	p.F1254L	0.99995	0.253	8.9265
MC_016_3	X-Linked	chrX:53082872-53082872:G:T	nonsynonymous SNV	*TSPYL2*	p.G125V	0.87393	0.405	3.1839
MC_017_3	X-Linked	chrX:130409298-130409298:A:C	nonsynonymous SNV	*RBMX2*	p.K72T	0.93892	0.337	2.7594
MC_019_3^a^	De novo	chr11:110137378-110137378:G:A	nonsynonymous SNV	*ZC3H12C*	p.R246K	0.99838	0.252	4.9736
MC_022_3^a^	De novo	chr14:90304095-90304095:C:T	stopgain	*NRDE2*	p.W282X	2.7953E−15	0.768	2.9904
MC_022_3^a^	X-Linked	chrX:3320746-3320746:C:G	nonsynonymous SNV	*MXRA5*	p.E1647Q	0.043013	0.398	5.1219
MC_024_3	Inherited homozygous	chr2:98822021-98822021:C:G	nonsynonymous SNV	*KIAA1211L*	p.R751P	0.99694	0.244	4.3461
MC_024_4	Inherited homozygous	chr1:109717382-109717382:C:T	stopgain	*GSTM5*	p.R205X	1.1942E−07	1.545	0.18805
MC_024_4	Inherited homozygous	chr2:209694400-209694400:G:A	nonsynonymous SNV	*MAP2*	p.D740N	1	0.105	6.9461
MC_025_3	Inherited homozygous	chr20:3045704-3045704:-:GCCCC	frameshift insertion	*GNRH2*	p.S116Rfs*11	1.3359E−07	1.918	−0.9844
MC_025_3	X-Linked	chrX:48059415-48059415:C:T	nonsynonymous SNV	*ZNF630*	p.G343R	0.000119	1.101	1.3081
MC_025_4	X-Linked	chrX:48059415-48059415:C:T	nonsynonymous SNV	*ZNF630*	p.G343R	0.000119	1.101	1.3081
MC_027_3	X-Linked	chrX:106206166-106206166:C:T	nonsynonymous SNV	*PWWP3B*	p.S245L	–	–	–
MC_032_3	Inherited homozygous	chr1:24652527-24652527:G:T	nonsynonymous SNV	*SRRM1*	p.K190N	1	0.146	6.3753
MC_034_3^a^	De novo	chr7:1746707-1746707:G:A	nonsynonymous SNV	*ELFN1*	p.R704Q	0.99882	0.153	4.1
MC_038_3	Inherited homozygous	chr19:47375602-47375602:G:-	frameshift deletion	*DHX34*	p.R734Pfs*38	6.5588E−13	0.768	2.8842
MC_039_3	X-Linked	chrX:108153670-108153670:G:T	nonsynonymous SNV	*ATG4A*	p.E323D	0.98953	0.262	3.7272
MC_042_3	X-Linked	chrX:111847345-111847345:G:A	nonsynonymous SNV	*TRPC5*	p.S490L	0.99973	0.17	4.7221
MC_043_3^a^	X-Linked	chrX:141907158-141907159:AG:-	frameshift deletion	*MAGEC1*	p.Q585Rfs*61	0.079896	1.913	−0.2768
MC_045_3	Compound heterozygous	chr14:31113108-31113108:A:G	nonsynonymous SNV	*HECTD1*	p.I2049T	1	0.158	9.8105
MC_045_3	Compound heterozygous	chr14:31121482-31121482:T:A	nonsynonymous SNV	*HECTD1*	p.Q1713H	1	0.158	9.8105
MC_045_3	X-Linked	chrX:107597521-107597521:G:A	nonsynonymous SNV	*FRMPD3*	p.E581K	0.00019028	0.476	4.6328
MC_047_3	De novo	chr19:12015095-12015095:-:C	frameshift insertion	*ZNF433*	p.E591Gfs*3	0.012227	1.691	0.50362
MC_050_3	Inherited homozygous	chr1:16958675-16958675:G:C	nonsynonymous SNV	*CROCC*	p.E1319D	1.2656E−24	0.71	3.9469
MC_051_4	De novo	chr4:176150054-176150054:-:TATA	stopgain	*WDR17*	p.E687Vfs*2	9.6547E−25	0.85	2.6234
MC_053_3	Inherited homozygous	chr6:47682450-47682450:C:T	stopgain	*ADGRF2*	p.R631X	–	–	–
MC_053_3	Inherited homozygous	chr9:18928536-18928548:GGGCATGTGTAAT:-	frameshift deletion	*SAXO1*	p.H245Lfs*58	–	–	–
MC_053_4	De novo	chr2:241690632-241690632:C:T	stopgain	*ING5*	p.R8X	0.61851	0.482	3.0217
MC_053_4	De novo	chr8:113314689-113314689:T:C	nonsynonymous SNV	*CSMD3*	p.I95V	0.057105	0.299	9.9645
MC_055_3^a^	De novo	chr19:53353471-53353472:TC:-	stopgain	*ZNF845*	p.H933*	0.00095913	1.9	−0.3907
MC_055_3^a^	De novo	chr10:26511804-26511804:C:T	nonsynonymous SNV	*APBB1IP*	p.R197W	0.85443	0.371	4.0617
MC_055_3^a^	De novo	chr17:42295746-42295746:C:T	nonsynonymous SNV	*STAT5A*	p.T138I	0.99994	0.202	5.6916
MC_055_3^a^	X-Linked	chrX:102653932-102653932:G:T	stopgain	*GPRASP1*	p.E7X	0.31099	0.416	4.2
MC_060_3	X-Linked	chrX:108137147-108137147:A:T	nonsynonymous SNV	*ATG4A*	p.N98I	0.98953	0.262	3.7272
MC_063_4	X-Linked	chrX:37009816-37009816:C:G	nonsynonymous SNV	*FAM47C*	p.T469S	–	–	–
MC_063_5	X-Linked	chrX:151671121-151671121:A:C	nonsynonymous SNV	*PASD1*	p.E385D	0.82307	0.379	3.9976
MC_063_5	X-Linked	chrX:37009816-37009816:C:G	nonsynonymous SNV	*FAM47C*	p.T469S	–	–	–
MC_066_3	De novo	chr7:50400256-50400256:T:C	nonsynonymous SNV	*IKZF1*	p.S167P	0.9986	0.156	4.0515
MC_077_3^a^	X-Linked	chrX:141896926-141896926:A:G	nonsynonymous SNV	*MAGEC3*	p.S92G	7.6566E−16	1.722	−0.8259
MC_077_3^a^	X-Linked	chrX:65502089-65502089:C:T	nonsynonymous SNV	*ZC3H12B*	p.S464L	0.99835	0.206	4.2592
MC_088_5	X-Linked	chrX:73563448-73563448:A:G	nonsynonymous SNV	*CHIC1*	p.E55G	0.85254	0.476	2.3226
MC_099_3^a^	De novo	chr1:241639950-241639954:CAGGA:-	frameshift deletion	*OPN3*	p.S101Pfs*18	0.0012801	0.894	1.8617
MC_100_4^a^	De novo	chr12:18562854-18562855:AG:-	frameshift deletion	*PIK3C2G*	p.R1207Sfs*12	9.5013E−40	1.136	0.60465
MC_101_3	X-Linked	chrX:136349791-136349791:G:T	nonsynonymous SNV	*ADGRG4*	p.V2029F	–	–	–
MC_106_4	De novo	chrX:152652679-152652679:A:G	nonsynonymous SNV	*GABRQ*	p.S433G	0.0042977	0.736	2.3834
MC_109_3	X-Linked	chrX:48058815-48058815:A:G	nonsynonymous SNV	*ZNF630*	p.Y543H	0.000119	1.101	1.3081
MC_113_3	Compound heterozygous	chr12:121233045-121233046:CT:-	frameshift deletion	*P2RX4*	p.Y339Lfs*7	4.9835E−12	1.282	0.54494
MC_113_3	Compound heterozygous	chr12:121229057-121229057:C:T	nonsynonymous SNV	*P2RX4*	p.T254I	4.9835E−12	1.282	0.54494
MC_115_4	X-Linked	chrX:27747991-27747991:A:G	nonsynonymous SNV	*DCAF8L2*	p.K366E	0.81931	0.474	2.6395
MC_116_4	De novo	chr3:98133091-98133091:T:C	nonsynonymous SNV	*OR5H1*	p.Y132H	4.6874E−05	1.874	−0.3953
MC_116_4	De novo	chr12:1646012-1646013:TG:-	frameshift deletion	*WNT5B*	p.V281Gfs*34	0.58535	0.494	2.9706
MC_117_3	X-Linked	chrX:151744343-151744343:C:T	nonsynonymous SNV	*CNGA2*	p.R614C	0.00098232	0.787	2.2373
MC_117_3	X-Linked	chrX:55003032-55003032:C:T	nonsynonymous SNV	*APEX2*	p.P165S	0.97302	0.268	3.0929
MC_117_3	X-Linked	chrX:143629851-143629851:C:T	nonsynonymous SNV	*SLITRK4*	p.V420I	0.79447	0.422	3.3262
MC_117_4	X-Linked	chrX:151744343-151744343:C:T	nonsynonymous SNV	*CNGA2*	p.R614C	0.00098232	0.787	2.2373
MC_117_4	X-Linked	chrX:55003032-55003032:C:T	nonsynonymous SNV	*APEX2*	p.P165S	0.97302	0.268	3.0929
MC_117_4	X-Linked	chrX:143629851-143629851:C:T	nonsynonymous SNV	*SLITRK4*	p.V420I	0.79447	0.422	3.3262
MC_120_3	Inherited homozygous	chr13:113084912-113084912:T:G	stopgain	*MCF2L*	p.Y656X	2.7178E−07	0.504	4.8643
MC_124_6	X-Linked	chrX:152991384-152991385:GT:-	frameshift deletion	*PNMA5*	p.T72Cfs*25	–	–	–
MC_125_4	X-Linked	chrX:37009783-37009783:G:T	nonsynonymous SNV	*FAM47C*	p.R458L	–	–	–
MC_129_5^a^	De novo	chr5:850480-850480:-:AA	frameshift insertion	*ZDHHC11*	p.Q42Sfs*4	1.1047E−15	1.541	−0.3378
MC_130_3^a^	Inherited homozygous	chr11:57380702-57380702:A:-	frameshift deletion	*PRG3*	p.C3Afs*28	0.00033439	1.143	1.2307
MC_135_3	X-Linked	chrX:53084603-53084603:G:A	nonsynonymous SNV	*TSPYL2*	p.R289H	0.87393	0.405	3.1839
MC_138_3	X-Linked	chrX:151743323-151743323:C:T	nonsynonymous SNV	*CNGA2*	p.R274C	0.00098232	0.787	2.2373
MC_138_3	X-Linked	chrX:107602115-107602115:C:T	nonsynonymous SNV	*FRMPD3*	p.S1392F	0.00019028	0.476	4.6328
MC_138_4	X-Linked	chrX:151743323-151743323:C:T	nonsynonymous SNV	*CNGA2*	p.R274C	0.00098232	0.787	2.2373
MC_138_4	X-Linked	chrX:107602115-107602115:C:T	nonsynonymous SNV	*FRMPD3*	p.S1392F	0.00019028	0.476	4.6328
MC_144_3^a^	De novo	chr6:70528348-70528348:G:A	nonsynonymous SNV	*FAM135A*	p.S995N	0.99893	0.265	6.2502
MC_144_3^a^	Inherited homozygous	chr19:54813219-54813219:-:A	frameshift insertion	*KIR2DL4;LOC112268354*	p.M271Nfs*108	–	–	–
MC_146_3	Inherited homozygous	chr6:78885392-78885393:TG:-	frameshift deletion	*IRAK1BP1*	p.V111Dfs*5	1.449E−06	1.398	0.55995
MC_146_3	X-Linked	chrX:53085669-53085669:A:G	nonsynonymous SNV	*TSPYL2*	p.D426G	0.87393	0.405	3.1839
MC_146_3	X-Linked	chrX:53085670-53085670:C:A	nonsynonymous SNV	*TSPYL2*	p.D426E	0.87393	0.405	3.1839
MC_149_3	X-Linked	chrX:15479743-15479743:G:A	stopgain	*PIR*	p.R59X	5.3402E−10	1.799	−0.6034
MC_149_3	X-Linked	chrX:3317781-3317781:G:T	nonsynonymous SNV	*MXRA5*	p.T1967N	0.043013	0.398	5.1219
MC_150_3	Compound heterozygous	chr12:131915950-131915950:G:A	nonsynonymous SNV	*ULK1*	p.A557T	0.99318	0.288	5.5149
MC_150_3	Compound heterozygous	chr12:131917030-131917030:C:A	nonsynonymous SNV	*ULK1*	p.T717K	0.99318	0.288	5.5149
MC_151_3	Inherited homozygous	chr7:151195598-151195598:C:-	frameshift deletion	*IQCA1L*	p.E459Kfs*4	–	–	–
MC_154_3^a^	De novo	chr12:113105758-113105776:GCCAGACGTAGCGCTTCTT:-	frameshift deletion	*RASAL1*	p.K589Sfs*17	1.2135E−14	0.953	1.7907
MC_156_3^a^	De novo	chr1:52274480-52274480:T:A	nonsynonymous SNV	*ZFYVE9*	p.F822Y	0.90244	0.325	5.5725
MC_156_3^a^	Inherited homozygous	chr17:16353365-16353372:GGGGGCCG:-	frameshift deletion	*CENPV*	p.A22Gfs*20	0.33194	0.736	2.0766
MC_156_3^a^	X-Linked	chrX:154716292-154716292:C:T	nonsynonymous SNV	*GAB3*	p.R37H	0.9536	0.341	3.5492
MC_158_3^a^	De novo	chr5:16694505-16694505:T:-	frameshift deletion	*MYO10*	p.G1225Afs*22	3.1562E−05	0.374	7.1688
MC_159_3	Compound heterozygous	chr6:7576422-7576422:C:T	nonsynonymous SNV	*DSP*	p.S920F	0.99978	0.26	8.6089
MC_159_3	Compound heterozygous	chr6:7579763-7579763:G:C	nonsynonymous SNV	*DSP*	p.E1191D	0.99978	0.26	8.6089
MC_159_3	Compound heterozygous	chr6:7579771-7579771:A:G	nonsynonymous SNV	*DSP*	p.K1194R	0.99978	0.26	8.6089
MC_159_3	De novo	chr1:20773459-20773459:C:T	nonsynonymous SNV	*HP1BP3*	p.A130T	0.98422	0.3	4.1625
MC_159_3	Inherited homozygous	chr5:112145946-112145946:A:T	stopgain	*EPB41L4A*	p.L662X	3.5133E−21	1.015	1.4643
MC_159_5	Inherited homozygous	chr5:112145946-112145946:A:T	stopgain	*EPB41L4A*	p.L662X	3.5133E−21	1.015	1.4643
MC_159_5	Inherited homozygous	chr14:70167414-70167414:C:A	stopgain	*SLC8A3*	p.E337X	0.00073391	0.592	3.3014
MC_160_3	De novo	chr5:144473681-144473681:C:T	nonsynonymous SNV	*KCTD16*	p.P285L	0.99329	0.2	3.5785
MC_160_3	De novo	chr3:124699950-124699950:A:T	nonsynonymous SNV	*KALRN*	p.Y940F	1	0.152	8.2551
MC_160_3	Inherited homozygous	chr4:682207-682222:TCCTGCTCCCCCTCGG:-	frameshift deletion	*SLC49A3*	p.D354Efs*22	–	–	–
MC_160_3	De novo	chrX:111776849-111776849:T:A	nonsynonymous SNV	*TRPC5*	p.S796C	0.99973	0.17	4.7221
MC_161_3^a^	De novo	chr19:56160179-56160179:T:G	nonsynonymous SNV	*ZNF444*	p.F320C	0.8667	0.454	2.3789
MC_161_3^a^	De novo	chr15:88527714-88527714:G:T	nonsynonymous SNV	*DET1*	p.L386I	0.67903	0.432	3.4607
MC_161_3^a^	De novo	chr2:158631756-158631756:-:A	frameshift insertion	*PKP4*	p.R387Tfs*6	5.269E−13	0.641	3.9671
MC_161_3^a^	De novo	chr1:226736927-226736927:A:G	nonsynonymous SNV	*ITPKB*	p.C178R	0.99994	0.148	5.0848
MC_161_3^a^	De novo	chr1:94174411-94174411:C:T	nonsynonymous SNV	*ARHGAP29*	p.D1018N	0.99964	0.249	6.0875
MC_161_3^a^	De novo	chr1:113973345-113973345:G:C	nonsynonymous SNV	*HIPK1*	p.V762L	1	0.121	6.4165
MC_161_3^a^	De novo	chr7:43492124-43492124:G:T	nonsynonymous SNV	*HECW1*	p.G1061V	0.99982	0.253	7.2151
MC_161_3^a^	Inherited homozygous	chr7:101557440-101557450:GGGTGGCGCCC:-	frameshift deletion	*COL26A1*	p.G413Tfs*38	2.9274E−06	0.832	2.2022
MC_164_3	Compound heterozygous	chr3:98132875-98132875:T:A	nonsynonymous SNV	*OR5H1*	p.Y60N	4.6874E−05	1.874	−0.3953
MC_164_3	Compound heterozygous	chr3:98132997-98132997:-:T	frameshift insertion	*OR5H1*	p.S103Ffs*19	4.6874E−05	1.874	−0.3953
MC_166_3^a^	De novo	chr1:30738992-30738992:C:G	nonsynonymous SNV	*LAPTM5*	p.S153T	0.90044	0.4	2.9211
MC_166_3^a^	De novo	chr3:195381926-195381926:A:C	nonsynonymous SNV	*ACAP2*	p.S70A	0.92325	0.324	5.4197
MC_166_3^a^	De novo	chr5:167375332-167375332:G:A	nonsynonymous SNV	*TENM2*	p.G121R	1	0.191	8.4851
MC_166_3^a^	De novo	chr3:68739137-68739137:T:A	stopgain	*TAFA4*	p.K117X	–	–	–
MC_166_3^a^	Inherited homozygous	chr10:115171344-115171345:TT:-	stoploss	*ATRNL1*	p.*468delinsIKSSYEF	0.99915	0.266	6.8065
MC_166_3^a^	X-Linked	chrX:16869523-16869523:G:A	nonsynonymous SNV	*RBBP7*	p.P40L	0.98393	0.28	3.5915
MC_170_3	X-Linked	chrX:152651603-152651603:G:T	nonsynonymous SNV	*GABRQ*	p.A327S	0.0042977	0.736	2.3834
MC_172_3^a^	De novo	chr2:219330807-219330807:G:-	frameshift deletion	*RESP18*	p.H101Ifs*25	8.716E−10	1.747	−0.4376
MC_172_3^a^	De novo	chr14:58353693-58353693:A:C	nonsynonymous SNV	*ARID4A*	p.E564A	1	0.139	7.0648
MC_172_3^a^	Inherited homozygous	chr1:120436639-120436640:AG:-	frameshift deletion	*NBPF8*	p.R41Mfs*20	1.4181E−07	1.967	−2.0676
MC_173_3	X-Linked	chrX:118975203-118975207:GGGGG:-	frameshift deletion	*LONRF3*	p.G142Rfs*14	0.41779	0.463	3.4438
MC_173_3	X-Linked	chrX:149594688-149594688:G:A	stopgain	*HSFX1;HSFX2*	p.R5X	–	–	–
MC_174_3	X-Linked	chrX:150671531-150671531:C:T	nonsynonymous SNV	*MTM1*	p.S546F	0.99961	0.176	4.6289
MC_175_3^a^	De novo	chr15:70679628-70679628:T:-	frameshift deletion	*UACA*	p.R278Gfs*16	3.2462E−29	0.973	1.7364
MC_175_3^a^	De novo	chr9:128735367-128735367:T:G	nonsynonymous SNV	*ZER1*	p.T640P	0.99992	0.178	5.2057
MC_175_3^a^	De novo	chr11:32934995-32934995:C:T	nonsynonymous SNV	*QSER1*	p.S1246F	0.99999	0.208	6.822
MC_175_3^a^	De novo	chr10:73792722-73792722:A:G	nonsynonymous SNV	*ZSWIM8*	p.D728G	1	0.133	7.7289
MC_175_3^a^	De novo	chr17:44356020-44356020:T:C	nonsynonymous SNV	*FAM171A2*	p.Y278C	0.90848	0.381	3.309
MC_175_3^a^	De novo	chr10:100487650-100487650:G:-	frameshift deletion	*SEC31B*	p.A1169Vfs*19	2.5856E−29	1.05	1.2242

All variants are exonic.

*LOEUF* loss-of-function observed/expected upper bound fraction.

^a^Samples with a missing parent sample where compound heterozygous variant calling was not possible and de novo, inherited homozygous, and X-linked variant calling relied on one parent only.

### Copy number variant analysis

Since CNVs are known to play an important role in ASD^26^, we analyzed CNVs in the ASD cohort. We called CNVs in individuals with ASD using individuals from the cohort who did not have ASD as controls, utilizing CNVkit^27^. In total, we identified 539 CNVs across all individuals with ASD, including 276 deletions and 263 duplications (Supplementary Data 9 and 10). The average size of a CNV was 243 kb, and there were 15 CNVs encompassing regions that did not include any genes. Out of the identified CNVs, 34 overlapped with known ASD CNVs as defined by the SFARI Gene database^21^, including the 3q29, 17p11.2, and 22q13.3 loci. Of the called CNVs, 23 also overlapped with syndromic CNVs from the DECIPHER database^28^. Some of these syndromes, such as Potocki-Lupski syndrome^29^ and Smith-Magenis syndrome^30^, are associated with neurodevelopmental phenotypes. Although our data demonstrate an overlap between CNVs and specific genomic regions, this does not imply that the CNVs are causal. Further investigation is needed to establish the pathogenicity of these variants.

## Discussion

We performed WES in a modest familial cohort consisting of 754 individuals from 195 families, with at least one child in each family diagnosed with ASD by a neurologist, child psychiatrist, or psychologist. It is important to note that the source of patient ascertainment can introduce bias; for example, recruitment through clinical centers may be skewed towards cases with comorbid conditions^31^. Furthermore, the difficulty in diagnosing ASD, particularly in patients with severe intellectual disability^32^, makes it challenging to determine whether the identified variants are exclusively associated with ASD or if they also contribute to broader neurodevelopmental disorders. The families enrolled in the cohort represented diverse ancestral backgrounds, including African American, Asian, Hispanic, Middle Eastern, Native American, and European. Sequencing a diverse cohort offered a broader genetic landscape, reduced bias, captured population-specific alleles, and provided wider global relevance. While our sample size limited in-depth ancestry-specific analyses^5,33^, future studies with larger samples can expand on this groundwork.

In total we discovered 38,834 novel private variants in the cohort that have not been previously reported. The lack of large public datasets for most of the ancestries represented in our cohort can affect the incidence of observed variants and could contribute to the number of novel private variants detected. We employed a variant filtration and prioritization pipeline that implements established practices in the field and aligns with other large-scale studies^6,7,34^, including implementing filtering strategies for all inheritance modes, utilizing deleteriousness prediction algorithms, and incorporating gene constraint scores. Due to the modest size of our cohort, we were unable to leverage more sophisticated methods like the Bayesian analysis framework. Our analysis identified 92 potentially pathogenic coding variants in 73 known neurodevelopmental disease genes. The known genes included ASD genes *BCORL1*, *CDKL5*, *MECP2*, and *SETD1B*, among other neurodevelopmental disease genes (e.g., *ADGRV1*, *ATP7A*, *CHD5*, and *SCN3A*). In addition, we compared our findings to data from large-scale cohorts^6^. Out of the 73 genes, we identified overlap with 11 high-confidence ASD genes identified by Fu et al.^6^, including *ARID1B*, *ATP1A3, AUTS2, DLG4*, *DYNC1H1*, *KMT2C*, *PLXNA1*, *SCN1A*, *SKI*, *SLC6A1*, and *SMARCA2*, strengthening our results. We also identified 158 potentially pathogenic coding variants in 120 candidate ASD genes (e.g., *DLG3*, *GABRQ*, *KALRN*, and *NCOR2*). For each of our candidate genes, we analyzed published data from Zhou et al.^7^ to obtain *P* values and transmission disequilibrium test (TDT) statistic values representing the contribution of de novo and rare inherited loss-of-function variants to ASD risk, respectively. Although the candidate genes did not reach study-wide significance by de novo variant enrichment (requiring *p* < 0.001), 4 of them—*ATF7IP, ATRNL1, HECTD1,* and *QSER1*—passed the Zhou et al.^7^ TDT filtering step (TDT statistic ≥ 1, within top 20% LOEUF, and A-risk ≥ 0.4). This is unsurprising, given the familial nature of the cohort in this study and the much larger case-control cohort in Zhou et al.^7^. In addition, 3 of the identified candidate genes—*CENPV*, *HECTD1*, and *MAP2*—overlapped with high-confidence neurodevelopmental disease genes reported by Fu et al.^6^.

Tables 2 and 3 summarize the variants we identified in each individual with ASD, specifically in known ASD and neurodevelopmental disease genes, as well as in new candidate genes, respectively. Our analysis revealed distinct sets of genes that merit further investigation. Out of 222 individuals with ASD, we identified at least one potentially pathogenic variant in 112 individuals (~50%), out of which 68 individuals have at least one potentially pathogenic variant in a known neurodevelopmental disease gene (~30%). One of the aims of this study was to aid in identifying causative variants in the probands. The broad phenotypic assessment of the probands limited the granularity of our phenotype-genotype correlations. Furthermore, complete phenotype information was not available for all probands. Nevertheless, our findings are consistent with previous reports on the association between mutations in the identified genes and the observed phenotypes in probands, with commonality in language impairment and developmental delay across variants and probands. For example, proband MC-005-3 presented with ASD, seizures, and learning disabilities, in line with phenotypes of patients with pathogenic *CDKL5* mutations^35^. *SETD1B* mutations have been associated with intellectual developmental disorder with seizures and language delay (MIM # 611055)^36–38^. Probands with variants in *SETD1B* presented with language impairment (MC-146-3, MC-166-3) and seizures (MC-146-3). For proband MC-124-6, our analysis identified a de novo stopgain mutation in *CHAMP1*. Mutations in this gene are associated with neurodevelopmental phenotypes, including impaired language and speech (MIM # 616327)^39^, all of which are present in the proband. MC-106-4 and MC-170-3 have variants in *GABRQ*, associated with essential tremor and ASD^40,41^. *DLG3* mutations were identified in MC-001-3 and MC-001-4, and have been associated with X-linked intellectual disability^42,43^. Other interesting genes included *HECTD1* (MC-045-3) and *HECW1* (MC-161-3), which encode proteins predicted to enable ubiquitin ligase activity^44^. *NCOR2* (with a variant daintified in JC-24-3) encodes a nuclear receptor co-repressor 2 that mediates transcriptional silencing of target genes by promoting chromatin condensation, thus preventing access to basal transcription machinery^45–47^. Sequencing studies in larger cohorts and additional experimental validation will be required to establish causality for the candidate genes that have not been previously linked to ASD.

In conclusion, by sequencing a diverse ASD cohort of individuals from over ten ancestries, this study breaks away from the limitations of single-population analyses and contributes to the ongoing effort of identifying causative genes and variants. While further functional validation is necessary to pinpoint causal variants in probands, these findings provide a valuable roadmap for more targeted future research, which will ultimately deepen our understanding of this spectrum of disorders.

## Methods

### Subjects and specimens

All human studies were reviewed and approved by the institutional review board (IRB) of the University of Texas Southwestern Medical Center (UTSW), the research committee at the University of Jordan School of Medicine, the ethics committee of the Jordan University Hospital, and the IRB of the Jordan University of Science and Technology. We have complied with all relevant ethical regulations, including the Declaration of Helsinki. Families were primarily recruited from the Dallas Fort Worth area, with some families recruited from Jordan, and written informed consent was obtained from all study participants. Inclusion criteria included a diagnosis of autism spectrum disorder (ASD) by a neurologist, child psychiatrist, or psychologist. Patients with genetically defined syndromes, specifically Fragile X syndrome, Angelman syndrome, Rett syndrome, or Tuberous sclerosis complex, were excluded from study participation. All patients enrolled in the study received a diagnosis of ASD from their referring clinicians, who performed physical and behavioral assessments and administered the following standard ASD diagnostic measures: (1) Autism Diagnostic Observation Schedule, Second Edition (ADOS-2)—a semi-structured, standardized assessment of communication, social interaction, play, and restricted and repetitive behaviors; (2) The Autism Diagnostic Interview-Revised (ADI-R)—this established assessment took ~1.5–3 h to administer, during which an experienced clinical interviewer interviewed a parent or caregiver familiar with the developmental history and current behavior of the individual being evaluated; (3) Diagnostic and Statistical Manual of Mental Disorders (DSM-V). Since the recruitment sources included multiple sites, there may be instances where not all three tests were performed. This, along with inter-site differences, may present potential sources of variance in our study. Blood samples were collected from all available family members by peripheral venipuncture and genomic DNA was isolated from circulating leukocytes using AutoPure (Qiagen, Hilden, Germany) according to the manufacturer’s instructions.

### Sample preparation and sequencing

All samples were prepared for sequencing using a custom automated sample preparation workflow developed at the Regeneron Genetics Center (RGC). Genomic DNA libraries were created by enzymatically shearing DNA to a mean fragment size of 200 base pairs using reagents from New England Biolabs. A common Y-shaped adapter (IDT) was ligated to all DNA libraries. Unique, asymmetric 10 base pair barcodes were added to the DNA fragments during library amplification with Kapa HiFi to facilitate multiplexed exome capture and sequencing. Equal amounts of sample were pooled prior to overnight exome/genotype capture with the Twist Comprehensive Exome panel, RGC developed Twist Diversity SNP panel, and additional spike-ins to boost coverage at selected CHIP sites and to cover the mitochondrial genome; all samples were captured on the same lot of oligos. The captured DNA was PCR amplified and quantified by qPCR. The multiplexed samples were pooled and then sequenced using 75 base pair paired-end reads with two 10 base pair index reads on the Illumina NovaSeq 6000 platform on S4 flow cells.

### Whole exome sequencing and data processing

Sequencing reads from both exome and genotyping assays in FASTQ format were generated from Illumina image data using bcl2fastq program (Illumina). Following the OQFE (original quality functional equivalent) protocol^48^, sequence reads were mapped to the human reference genome version GRCh38 using BWA MEM^49^ in an alt-aware manner, read duplicates were marked, and additional per-read tags were added. For exome data, single nucleotide variants (SNVs) and short insertions and deletions (indels) were identified using a Parabricks accelerated version of DeepVariant v0.10 with a custom WES model and reported in per-sample genome variant call format (gVCF) files. These exome gVCFs were aggregated with GLnexus v1.4.3 using the pre-configured DeepVariantWES setting^50^ into joint-genotyped multi-sample project-level VCF (pVCF), which was converted to bed/bim/fam format using PLINK 1.9^51^. Depth was calculated using mosdepth^52^ and coverage was assessed using custom scripts. The percent coverage was calculated as the number of base pair positions sequenced to a given depth divided by the total number of bases sequenced.

VCF files for SNVs and indels were annotated with ANNOVAR^53^ using allele frequencies from the 1000 Genomes project (1000G)^12^, the Genome Aggregation Database (gnomAD)^16,17^, the Greater Middle East Variome project (GME)^18^, and the Exome Aggregation Consortium (ExAC)^19^. The variants were also annotated using the Single Nucleotide Polymorphism Database (dbSNP)^54^, the database of Human Non-synonymous SNVs and Their Functional Predictions and Annotations (dbNSFP)^55^, and ClinVar^56^. Annotated VCF files were uploaded into an SQL database for working storage and analysis. Exome data was stored, and analyses were performed on the Texas Advanced Computing Center (TACC) high-performance computing servers, a resource of the University of Texas (Austin, TX).

### Variant filtration

Variants having a read depth of ≥ 10 and a genotype quality (GQ) score of ≥ 30 were retained as quality filtered. Rare variants were defined as those with minor allele frequencies (MAF) < 1% in 1000G^12^, gnomAD v2.1^16,17^, GME^18^, and ExAC^19^. When filtering for rare variants, we used the overall population frequency data from the previously mentioned databases. We further refined the analysis by applying the same cutoffs to each sub-population within the dataset as well. Novel variants were defined as variants that are not found in the four aforementioned public datasets. Private variants were defined as novel variants that occurred only in a single individual in our cohort. De novo variants were defined as heterozygous private variants present in individuals with ASD (absent from the exome of the father, the mother, and the sibling(s) when available). To minimize potential false positive de novo calls, we applied additional filtering steps, requiring that de novo variants have the following criteria: (1) GQ ≥ 99, (2) alternate allele depth (AD-Alt) ≥ 10, (3) reference allele depth (AD-Ref) ≥ 10, (4) 0.3 ≤ AD-Alt/read depth (DP) ≤ 0.7, (5) Allele Quality score ≥ 999, (6) length(Alt) ≤ 50 and length(Ref) ≤ 50. Compound heterozygous variants in offspring were defined as inherited heterozygous variants that occurred within the same gene and that were present in heterozygous form in one parent but not the other. All compound heterozygous variants were filtered for AD-Alt ≥ 10, AD-Ref ≥ 10, and 0.3 ≤ AD-Alt/DP ≤ 0.7. Inherited homozygous variants were required to be present in heterozygous form in both the father and the mother, excluding variants that are homozygous in either one of the parents or siblings with no ASD when available, on the assumption of full penetrance. X-linked variants were X chromosome-specific and were required to be present in a male offspring and heterozygous in the mother.

### Variant prioritization

Rare variants that are de novo, compound heterozygous, inherited homozygous, or X-linked, were considered to be possibly damaging if they met the following criteria: (1) splice site variants, (2) exonic variants with a predicted protein effect of frameshift indels, nonframeshift indels, stopgain, stoploss, or unknown effect, (3) exonic nonsynonymous SNVs that were predicted to be damaging by at least 1 of the 2 algorithms used: SIFT^57,58^ and PolyPhen-2 HumVar^59^. PolyPhen-2 HumVar was chosen over PolyPhen-2 HumDiv because the former is more appropriate for Mendelian variants with drastic effect as we expect for ASD, while the latter is appropriate for common variants of smaller effect size. Possibly damaging variants were compared to the list of genes implicated in ASD from the Simons Foundation Autism Research Initiative (SFARI) Gene 2018 database (using the 2023 Q2 release)^21^. Variants were also screened for any phenotypic association in the Online Mendelian Inheritance in Man (OMIM) database^60^. Gene constraint was assessed using pLI, LOEUF, and Z scores from gnomAD v2.1^16,17^. To help assess a variant’s potential pathogenicity, the variants were also annotated with ClinVar data and the number of homozygous carriers in gnomAD v4.1^16,17^. To prioritize candidate disease variants (potentially pathogenic variants), we performed the following steps: (1) If the exact same variant was present in more than one unrelated person, it was excluded; (2) Variants within genes that had a SFARI Gene^21^ score of 1, 2, or S, or were associated with a neurological phenotype as annotated by OMIM were considered as “known” and the rest were considered as “novel”; (3) Within the “known” and “novel” lists, genes having multiple different variants in different people were prioritized; (4) We prioritized loss-of-function (LoF) variants and nonsynonymous SNVs with high probability of deleteriousness based on scores from prediction tools, including SIFT, PolyPhen-2 HumVar, VEST^61,62^, CADD^63^, and phyloP^64^; (5) We prioritized variants within genes with higher pLI (> 0.5) and lower LOEUF (< 0.5) scores. Steps 3-5 were performed sequentially, therefore, a variant was not required to satisfy all subsequent steps if it passed the initial ones; (6) We filtered out variants with ClinVar significance value as benign or likely benign; (7) We filtered out variants having one or more homozygous carriers in gnomAD v4.1^16,17^. The gene *TTN* is classified as an ASD gene in the SFARI Gene database^21^ with a score of 2. However, due to the large size of *TTN* (coding sequence of 108 kb), we calculated the missense mutation rate for *TTN* in each of the five probands with prioritized *TTN* variants (JC-21-3, JC-33-3, MC-014-3, MC-053-3, and MC-061-4) to account for its size. This rate was determined by dividing the total number of base pairs carrying missense mutations in *TTN* in each proband by the total length of the *TTN* coding region. Subsequently, we compared this ratio for each proband to the *TTN* missense mutation rate obtained from gnomAD v4.1^16,17^ (1.23 × 10^−5^). We found that the *TTN* missense mutation rate in each of the 5 probands (1.57 × 10^−4^, 2.50 × 10^−4^, 2.78 × 10^−4^, 3.33 × 10^−4^, and 3.70 × 10^−4^, respectively) exceeded the gnomAD rate. Consequently, we filtered out the *TTN* variants from the list of prioritized variants in “known” genes, but they are retained in the list of potentially damaging coding variants (Supplementary Data 5).

Since we observed more than one potentially pathogenic variant (in known and/or novel genes) in some probands, we also ranked them according to their likelihood of causing the disease in the proband. We followed the guidelines issued by the American College of Medical Genetics and Genomics and the Association for Molecular Pathology for the Interpretation of Sequence Variants^65^. We prioritized variants in known genes over novel genes. Stopgain/stoploss and frameshift variants were ranked over nonsynonymous SNVs, and de novo variants were ranked over other inherited variants. We also annotated the variants with AlphaMissense scores^66^ and prioritized those with higher scores.

### Copy number variant (CNV) analysis

We used CNVkit^27^ to detect CNVs based on the read depth in ASD samples relative to the average read depth in non-ASD samples in the cohort, using default parameters. Sample MC-064-3 was deemed as an outlier and removed from further analysis for having an unusually high number of CNVs (174 CNVs). The CNV calls segmentation file was filtered to include variants with *p* < 0.05 and copy number = 0, 1, 3, or 4. Variants were considered deletions if their log2 read depth ratio between the sample and control was ≤ −0.5. Variants were considered duplications if their log2 read depth ratio was ≥ 0.5. If the exact same CNV existed in more than one unrelated proband, it was filtered out. The filtered variants were annotated with known SFARI Gene^21^ CNVs and DECIPHER^28^ CNVs. The gnomAD structural variants v4.1^16,17^ frequencies were used to filter out common CNVs with a frequency >1% if the detected CNV completely overlapped with the gnomAD structural variant.

### Burden analysis

Nondisrupting variants were defined as exonic synonymous SNVs or exonic nonframeshift indels. The burden of rare LoF and predicted damaging missense variants was analyzed by comparing categories of variants identified in ASD versus non-ASD samples. LoF variants were defined as variants that are exonic or splice site predicted to result in a frameshift indel, a stopgain or stoploss, or splicing error. Missense variants were defined as nonsynonymous exonic or splice site. Missense damaging variants were defined as nonsynonymous SNVs that were predicted to be damaging by at least 1 of the 2 algorithms used: SIFT and PolyPhen-2 HumVar. Comparisons were made between ASD and non-ASD exomes in the above categories for all rare variants.

### Principal component analysis

Principal component analysis (PCA) was carried out in PLINK version 1.9^67^ using Phase 3 1000G^12^ data (populations shown in Supplementary Data 11). PCA input files from our samples were pruned for variants in linkage disequilibrium (LD) with an r2 > 0.2 in a 50 kb window. The LD-pruned dataset was generated using plink –indep-pairwise flag to compute the LD variants. Variants with chromosome mismatches, position mismatches, possible allele flips, and allele mismatches were identified and filtered out. The set of variants that remained was extracted from the 1000 G^12^ dataset and these were merged with our cohort dataset. PCA was run in PLINK using the –pca flag and the first two principal components were plotted in R. Analysis was performed for the entire cohort, pedigree founders, and probands.

### Specific expression analysis

We performed specific expression analysis (SEA) with human transcriptomics data from the BrainSpan collection^20^ to identify particular human brain regions and/or developmental windows potentially related to ASD pathophysiology along with candidate genes identified in individuals with ASD in this study. For each cell type or brain region, transcripts specifically expressed or enriched were identified at a specificity index (pSI) threshold of pSI < 0.05^68^. These analyses were performed using the Dougherty lab server (http://genetics.wustl.edu/jdlab/). Lists of candidate genes that overlapped with lists of transcripts enriched in a particular cell type or brain region were finalized using Fisher’s exact test with Benjamini–Hochberg correction. The significance level was set at *Q*-value < 0.05.

## Supplementary information

Supplemental Material

Supplemental Data

## Data Availability

Data are available in the main text or the Supplementary Materials. The whole exome sequencing data generated in this study are accessible through the database of Genotypes and Phenotypes (dbGaP) (accession number phs003603.v1.p1). Any additional information required to reanalyze the data reported in this paper is available from the corresponding author upon request. This study did not generate new unique reagents.

## Code availability

The code used for data analysis in this study is described in the Materials and Methods and is available on the Chahrour lab GitHub repository at: https://github.com/chahrourlab/WES.
