# Author Correction: The genetic landscape of autism spectrum disorder in an ancestrally diverse cohort

**DOI:** 10.1038/s41525-024-00458-0

**Published:** 2024-12-26

**Authors:** Ashlesha Gogate, Kiran Kaur, Raida Khalil, Mahmoud Bashtawi, Mary Ann Morris, Kimberly Goodspeed, Patricia Evans, Maria H. Chahrour

**Affiliations:** 1 Eugene McDermott Center for Human Growth and Development, University of Texas Southwestern Medical Center, Dallas, TX 75390 USA; 2 Department of Biotechnology and Genetic Engineering, Faculty of Science, University of Philadelphia, Amman, Jordan; 3 Department of Psychiatry, Jordan University of Science and Technology, King Abdullah University Hospital, Ramtha, Jordan; 4 UT Southwestern and Children’s Health Center for Autism Care, Children’s Medical Center Dallas, Dallas, TX 75247 USA; 5 Department of Pediatrics, University of Texas Southwestern Medical Center, Dallas, TX 75390 USA; 6 Department of Neurology, University of Texas Southwestern Medical Center, Dallas, TX 75390 USA; 7 Department of Psychiatry, University of Texas Southwestern Medical Center, Dallas, TX 75390 USA; 8 Department of Neuroscience, University of Texas Southwestern Medical Center, Dallas, TX 75390 USA; 9 Center for the Genetics of Host Defense, University of Texas Southwestern Medical Center, Dallas, TX 75390 USA; 10 Peter O’Donnell Jr. Brain Institute, University of Texas Southwestern Medical Center, Dallas, TX 75390 USA

**Keywords:** Autism spectrum disorders, Medical genomics

**Correction to:** *npj Genomic Medicine* 10.1038/s41525-024-00444-6, published online 04 December 2024

The wrong Supplementary file was originally published with this article; it has now been replaced with the correct. The original article has been corrected.

