# Supplementary material for: The genetic landscape of autism spectrum disorder in an ancestrally diverse cohort
Source: NPJ Genom Med. 2024 Dec 4;9:62. doi: 10.1038/s41525-024-00444-6 (PMC11618689; doi:10.1038/s41525-024-00444-6)
Supplement: Supplementary file 1 — Supplemental Material [file 41525_2024_444_MOESM1_ESM.pdf]

THE GENETIC LANDSCAPE OF AUTISM SPECTRUM DISORDER IN AN ANCESTRALLY DIVERSE COHORT

Ashlesha Gogate[1], Kiran Kaur[1], Raida Khalil[2], Mahmoud Bashtawi[3], Mary Ann Morris[4], Kimberly Goodspeed[4,5,6,7], Patricia Evans[4,5,6,7], Maria H. Chahrour[1,7,8,9,10] *

SUPPLEMENTARY MATERIALS

Supplementary Figures 1 to 6

Supplementary Data 1 to 11

SUPPLEMENTARY FIGURES

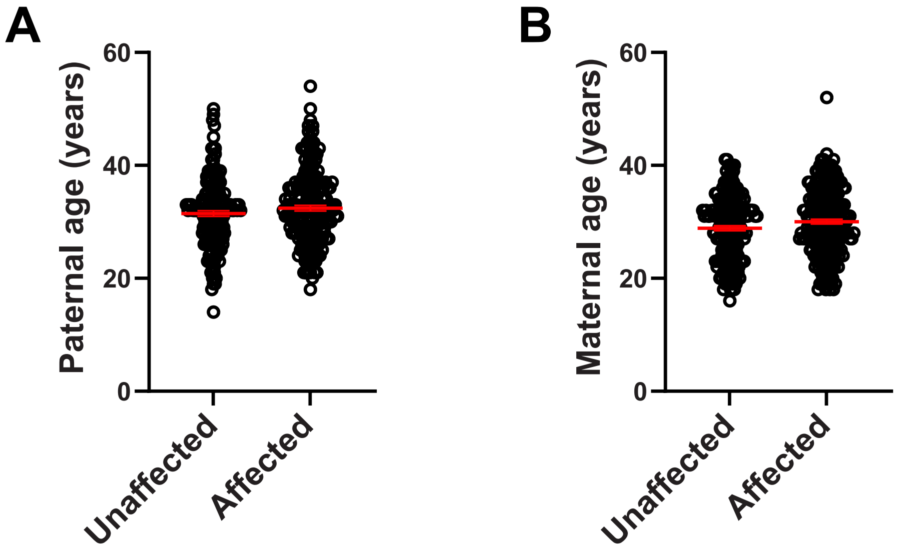

Supplementary Figure 1. Parental age in the ASD cohort. Paternal **(A)** and maternal **(B)** ages were plotted comparing the parental ages at the time of birth of offspring without ASD and offspring with ASD. There were no significant differences in the mean age of fathers or mothers at the time of birth of offspring without ASD (31 years for fathers, 29 years for mothers) compared to offspring with ASD (32 years for fathers, 30 years for mothers). Mean ± SEM are shown in red (paternal age: n=147 No ASD, 182 ASD, *P*=0.3765; maternal age: n=168 No ASD, 210 ASD, *P*=0.6820). Data were analyzed using unpaired t test.

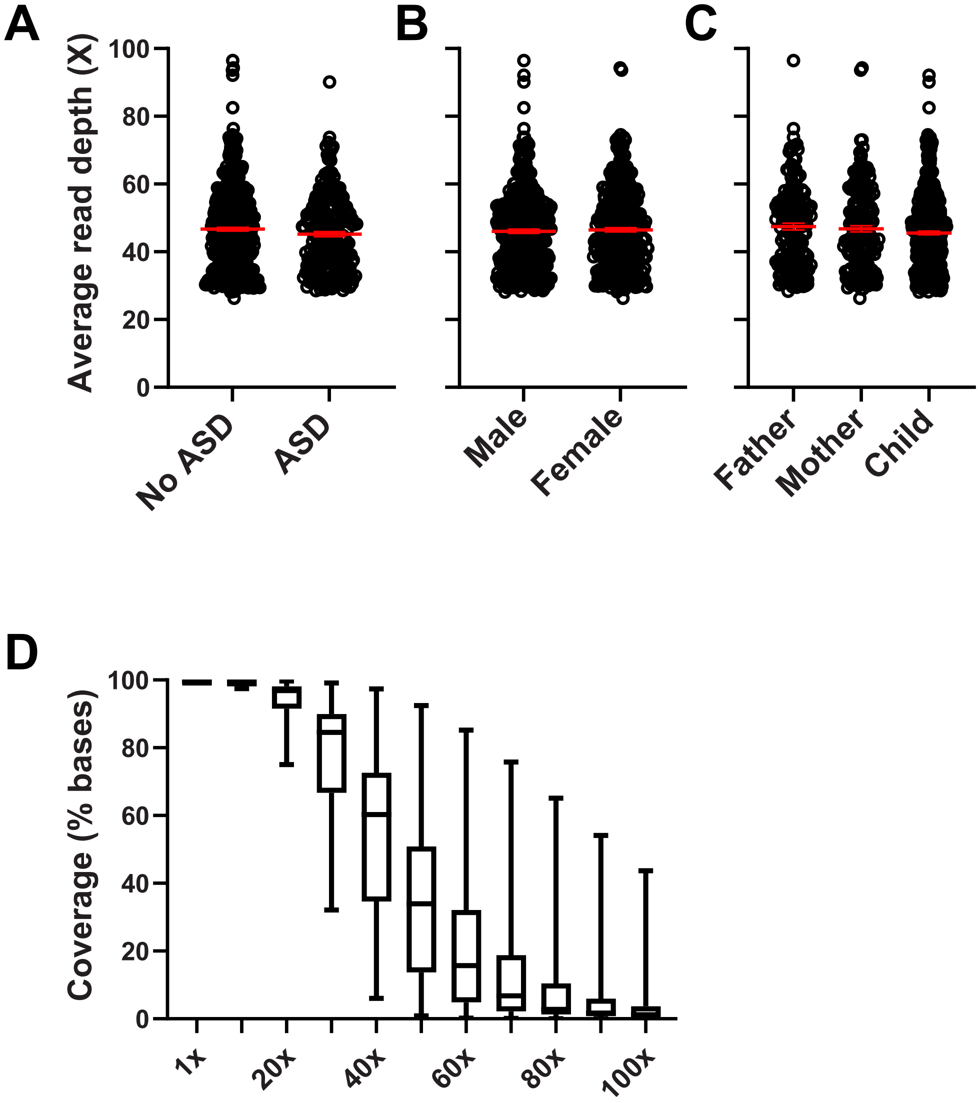

Supplementary Figure 2. Whole exome sequencing coverage statistics. The average read depth per sample was calculated across the cohort (mean ± SEM are shown in red). There were no significant differences in read depth across phenotypic status (*P*=0.1273) **(A)**, sex (*P*=0.6512) **(B)**, or family membership (*P*=0.2049) **(C)**. The average read depth was 47X for samples without ASD, 45X for samples with ASD, 46X for males, 46X for females, 47X for fathers, 47X for mothers, and 46X for children. Data were analyzed using unpaired t test for **(A)** and **(B)**, and one-way ANOVA for **(C)**. **(D)** The percentage of genomic bases covered at 1X, 10X, 20X, 30X, 40X, 50X, 60X, 70X, 80X, 90X, and 100X read depth was calculated for all samples across the cohort. At each value, the average, minimum, and maximum are plotted. On average, 99.29% and 93.9% of bases were covered at a mean read depth of at least 10X and 20X, respectively.

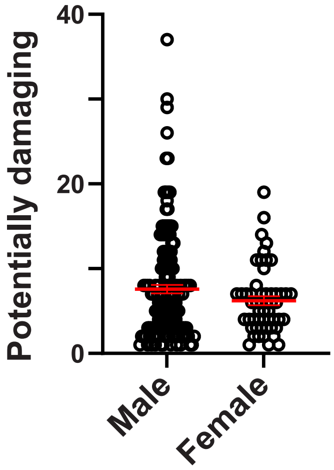

Supplementary Figure 3. Potentially damaging variants specific to the ASD cohort. The number of potentially damaging variants was compared for male versus female offspring with ASD. Potentially damaging variants are a subset of rare coding variants that are also predicted to be damaging. There was no significant difference in the prevalence of potentially damaging variants between male and female offspring with ASD (*P*=0.1362). Mean ± SEM are shown in red. Data were analyzed using unpaired t test.

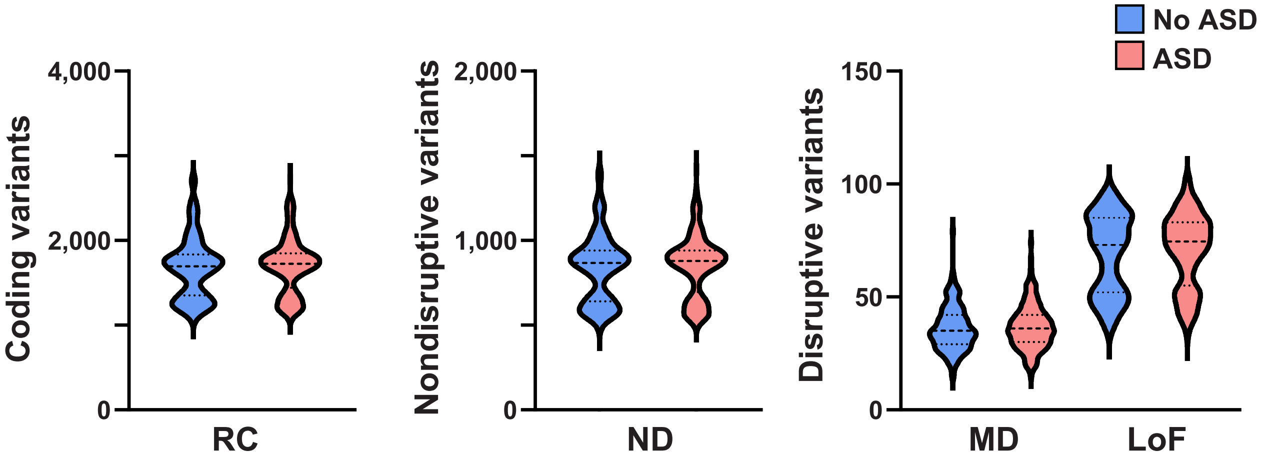

Supplementary Figure 4. Burden analysis of rare coding variants in the ASD cohort. Variant counts in samples without ASD versus samples with ASD are compared for all rare coding (RC) (*P*=0.4248), nondisrupting (ND) (*P*=0.4263), missense damaging (MD) (*P*=0.5099), and loss-of-function (LoF) (*P*=0.2652) variants. Data were analyzed using unpaired t test. Dotted lines denote 25%, 50%, and 75% marks.

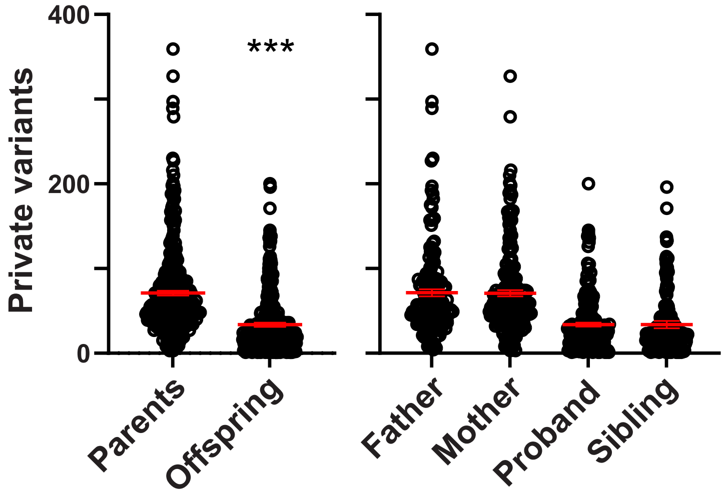

Supplementary Figure 5. Private variants specific to the ASD cohort. The number of private variants was compared for parents versus offspring (left) and by family membership (right). Private variants were defined as variants not previously annotated in population data from 1000G, gnomAD, GME, or ExAC, and also not present in other individuals in the cohort. Offspring had significantly fewer private variants (****P*<0.0001) compared to parents. There was no significant difference in the prevalence of private variants between offspring with ASD and siblings without ASD (*P*=0.9879) or between fathers and mothers (*P*=0.8976). Mean ± SEM are shown in red. Data were analyzed using unpaired t test.

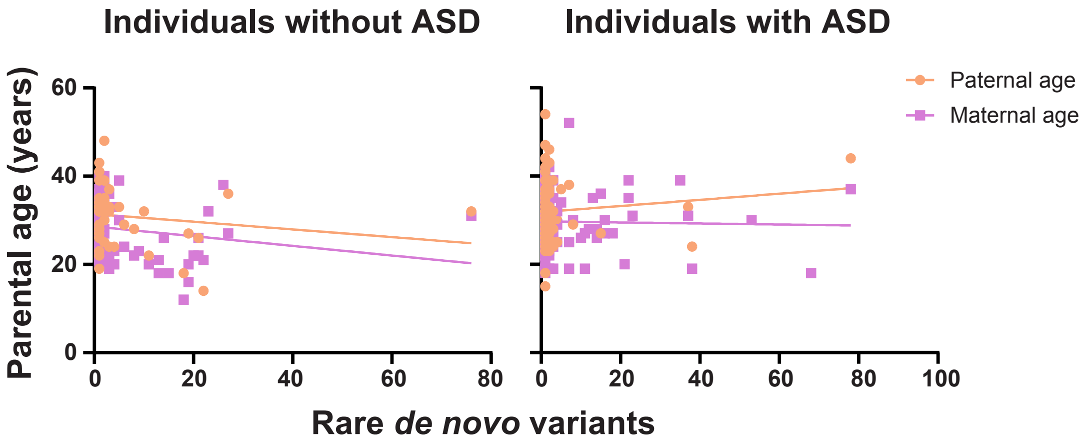

Supplementary Figure 6. The number of rare *de novo* variants with parental age in the ASD cohort. Linear regression analysis showed there were no significant changes in the number of rare *de novo* variants in individuals with ASD with paternal (P=0.3851, r^2=0.0116) or maternal (P=0.8197, r^2=0.0006) age at birth of the offspring with ASD and offspring without ASD (paternal age P=0.2486 and r^2=0.025, maternal age P=0.1262 and r^2=0.035).

SUPPLEMENTARY DATA LEGENDS

Supplementary Data 1. Demographics and clinical information for the ASD cohort. Age refers to current age in 2024. ADHD, Attention deficit/hyperactivity disorder; ASD, Autism spectrum disorder; DD, Developmental delay; ID, Intellectual disability; LD, Learning disability; OCD, Obsessive-compulsive disorder.

Supplementary Data 2. Variants identified by whole exome sequencing in the ASD cohort. Variant counts by individual for total variants called, single nucleotide variants (SNVs), insertions or deletions (Indels), quality filtered variants (QF), rare variants (defined as QF and having a MAF < 1% in 1000G, gnomAD, GME, and ExAC), rare heterozygous variants, rare homozygous variants, and novel variants (defined as previously unreported variants that have not been observed in any of the aforementioned datasets).

Supplementary Data 3. Private variants that are specific to the ASD cohort. Counts of variants by individual that have not been previously annotated in 1000G, gnomAD, GME, or ExAC. The variants are private to each individual, meaning not seen in other individuals in the cohort. Counts are given for private variants that are designated as possibly damaging by either of the used prediction tools (SIFT, PolyPhen-2 HumVar) in ASD and non-ASD individuals.

Supplementary Data 4. Variants that segregate with ASD identified by whole exome sequencing in individuals with ASD from the cohort. Variant counts by individual with ASD for rare quality filtered variants defined as having a MAF < 1% in all populations (1000G, gnomAD, GME, and ExAC) that are *de novo*, inherited homozygous, compound heterozygous, or X-linked. * Samples with a missing parent sample where compound heterozygous variant calling was not possible and *de novo*, inherited homozygous, and X-linked variant calling relied on one parent only.

Supplementary Data 5. Rare potentially damaging coding variants identified in individuals with ASD from the cohort. For compound heterozygous variants, the parent from which the allele was inherited is denoted in the "Parent" column. For SFARI score, S denotes syndromic genes. Indel, Insertion or deletion; SNV, Single nucleotide variant. * Samples with a missing parent sample where compound heterozygous variant calling was not possible and *de novo*, inherited homozygous, and X-linked variant calling relied on one parent only.

Supplementary Data 6. Specific expression analyses across brain regions. Analyses were performed using the genes associated with potentially pathogenic variants detected in individuals with ASD in the cohort. Each column lists the genes specifically expressed in a particular adult brain region. The values enclosed within parenthesis beside each brain region are Benjamini-Hochberg corrected P values for pSI (specificity index < 0.05).

Supplementary Data 7. Gene ontology (GO) analysis on known and candidate neurodevelopmental disease genes performed using DAVID.

Supplementary Data 8. Potentially pathogenic variants identified in each individual with ASD ranked according to their likeliness of causing disease in the proband. * Samples with a missing parent sample where compound heterozygous variant calling was not possible and *de novo*, inherited homozygous, and X-linked variant calling relied on one parent only.

Supplementary Data 9. The number of copy number variants (CNVs), deletions, and duplications detected per individual with ASD in the cohort. CNVs in individuals with ASD that overlap with CNVs that have been previously reported in ASD, defined as present in SFARI Gene, and other diseases as defined in DECIPHER, are also reported.

Supplementary Data 10. Copy number variants (CNVs) detected per individual with ASD in the cohort. gnomAD frequencies of the variants, CNVs in individuals with ASD that overlap with CNVs that have been previously reported in ASD, defined as present in SFARI Gene, and other diseases as defined in DECIPHER, are also reported. DEL, Deletion; DUP, Duplication.

Supplementary Data 11. The 1000 Genomes project populations. Abbreviations for the 1000G populations that were used for analyses are defined. Information was obtained from the 1000G website. AFR, African; AMR, Admixed American; EAS, East Asian; EUR, European; SAS, South Asian.
